# Genetic regulatory networks for salt-alkali stress in *Gossypium hirsutum* with differing morphological characteristics

**DOI:** 10.1186/s12864-019-6375-9

**Published:** 2020-01-06

**Authors:** Yanchao Xu, Richard Odongo Magwanga, Xiu Yang, Dingsha Jin, Xiaoyan Cai, Yuqing Hou, Yangyang Wei, Zhongli Zhou, Kunbo Wang, Fang Liu

**Affiliations:** 1 Zhengzhou Research Base, State Key Laboratory of Cotton Biology, Zhengzhou University/ Institute of Cotton Research, Chinese Academy of Agricultural Science, Zhengzhou, China; 2 School of Biological and Physical sciences (SBPS), Main campus, Jaramogi Oginga Odinga University of Science and Technology (JOOUST), P.O Box 210-40601, Bondo, Kenya; 3 Anyang Institute of Technology, Anyang, China

**Keywords:** Alkali-salt stress, RNA-Seq, Gene co-expression, *Gossypium hirsutum* races, WGCNA

## Abstract

**Background:**

Cotton grows in altering environments that are often unfavorable or stressful for its growth and development. Consequently, the plant must cope with abiotic stresses such as soil salinity, drought, and excessive temperatures. Alkali-salt stress response remains a cumbersome biological process and is regulated via a multifaceted transcriptional regulatory network in cotton.

**Results:**

To discover the molecular mechanisms of alkali-salt stress response in cotton, a comprehensive transcriptome analysis was carried out after alkali-salt stress treatment in three accessions of *Gossypium hirsutum* with contrasting phenotype. Expression level analysis proved that alkali-salt stress response presented significant stage-specific and tissue-specific. GO enrichment analysis typically suggested that signal transduction process involved in salt-alkali stress response at SS3 and SS12 stages in leaf; carbohydrate metabolic process and oxidation-reduction process involved in SS48 stages in leaf; the oxidation-reduction process involved at all three phases in the root. The Co-expression analysis suggested a potential *GhSOS3/GhCBL10-SOS2* network was involved in salt-alkali stress response. Furthermore, Salt-alkali sensitivity was increased in *GhSOS3* and *GhCBL10* Virus-induced Gene Silencing (VIGS) plants.

**Conclusion:**

The findings may facilitate to elucidate the underlying mechanisms of alkali-salt stress response and provide an available resource to scrutinize the role of candidate genes and signaling pathway governing alkali-salt stress response.

## Background

Allotetraploid Upland cotton (AD)_1_ (*G. hirsutum* L.) is an economically important crop around the world, providing the most natural fiber for the textile industries [1]. With the development of crop production and change in environment conditions, temperatures are extreme coupled with low precipitation, thus cotton plant must cope with various abiotic stress such as salinity, drought, and extreme temperatures. Salt-alkalization is a major global environmental and land resource issue [2]. Soil salinization and alkalization, frequently occurring together, significantly reduce crop productivity [3]. Moreover, soil salinization is a problem across the continents with the exception of un-assessed Antarctica [4]. Salinized soil is widely distributed all over the word, and occurs mainly on the waterfront, arid and semi-arid zones of more than 100 countries and regions worldwide [5]. According to statistics done by the United Nations Educational, Scientific and Cultural Organization (UNESCO), and the Food and Agriculture Organization (FAO) in 2005, Over 800 million hectares of land throughout the world are salty, in which 397 and 434 million hectares are affected through salinity and sodicity, respectively.).

Cotton is one important pioneer of salinized soil crop [6]. China is one of the largest cotton producing and cotton consuming countries in the world [1]. Currently, Xinjiang province is the largest cotton producing area in China [4]. Unfortunately, until now, although soil salt-alkalization could lead to crop growth damage and a decrease in crop yields, little attention has been paid on the molecular mechanisms of plant adaptation to alkaline stress [7]. But, many of scientists have greatly promoted the understanding of regulation mechanism of salt stress response in plant [8]. Salt-Overly-Sensitive (SOS) pathway was the first abiotic stress response signal pathway in plant [9]. With its further study of SOS pathway, more and more elements of this pathway were identified. In this pathway, cytosolic calcium signal was sensed by the EF-hand calcium-binding protein (SOS3) under salt stress. And then, SOS3 interacts with and activates SOS2, a serine/threonine protein kinase [10]. In a previous study, SOS3 is preferentially expressed in the root, and an SOS3 paralog, SCaBP8/CBL10 mainly expressed in the shoot, performs an equivalent role as SOS3 [11]. Additionally, A number of stress response genes have been identified in plants, including ion transporters, free radical scavengers, aquaporin ns, heat shock proteins, and late embryogenesis abundant proteins [12]. Such as, co-transformed the *GsPPCK3* and *SCMRP* genes into alfalfa demonstrated that the transgenic alfalfa displayed higher alkali tolerance [7]. And there is a research showed the physiologic mechanisms of enhanced resistance to alkali stress may be associated with increased antioxidant activity (via MDA, POD, SOD, and GSH), enhanced production of organic acids, and accumulation of osmolytes such as proline [13]. *OsCu/Zn-SOD* gene overexpression enhanced saline–sodic stress tolerance of *O. sativa* via increasing the detoxification capacity of reactive oxygen species in *O. sativa* and reducing salt-induced oxidative damage [14]. *SsMT2* gene, from alkaline-tolerant Suaeda salsa, plays an important role in reactive oxygen species scavenging and confers enhanced metal and oxidant tolerance to plants [15]. A superior tolerance wheat sample to alkaline stress conditions, is due to its strong absorbing ability for nutrient ions, a strong regulating ability for intracellular and rhizosphere pH and a more active reactive oxygen species (ROS) scavenging ability [16]. With the development of next generation sequence technology (NGS), more and more sequencing data were used for revealing the regulation mechanism of complex traits [17]. The weighted correlation network analysis (WGCNA) is an R package for gene co-expression network (GCN) analysis and can be used as a data exploration tool or a genetic screening (ranking) method to find clusters (modules) of highly correlated genes [18]. It was used widely to find hub genes in biomedical science [19–21]. In this study, transcriptome data were obtained by RNA-Seq of leaves and roots of three different upland cotton seedlings at various stages after salt-alkali stress treatment. A comparative transcriptome analysis was performed in three accessions of upland cotton with contrasting phenotypes under the salt-alkali stress. Transcriptomic data were dissected to show transcriptome dynamics and transcriptional network associated with different tissues/stages. The networks/modules of co-expressed genes expressed specifically at different tissues/stages after stress treatment of upland cotton were positively identified. GO enrichment analysis typically suggested that signal transduction process involved in salt-alkali stress response at SS3 and SS12 stages in leaf; carbohydrate metabolic process and oxidation-reduction process involved at SS48 stages in leaf; the oxidation-reduction process involved at all three phases in the root. Our ultimate result of *hub* gene analysis revealed precisely that *SOS3*/*CBL10-SOS2* network plays a core role in specific regulation of salt-alkali stress in upland cotton. This comprehensive study provides insights into the molecular mechanisms underlying salt-alkali stress response.

## Results

### Oxidant and antioxidant evaluation of the three upland cotton accessions CRI12, LAT40 and MAR85 under salt stress conditions

**Fig. 1:**
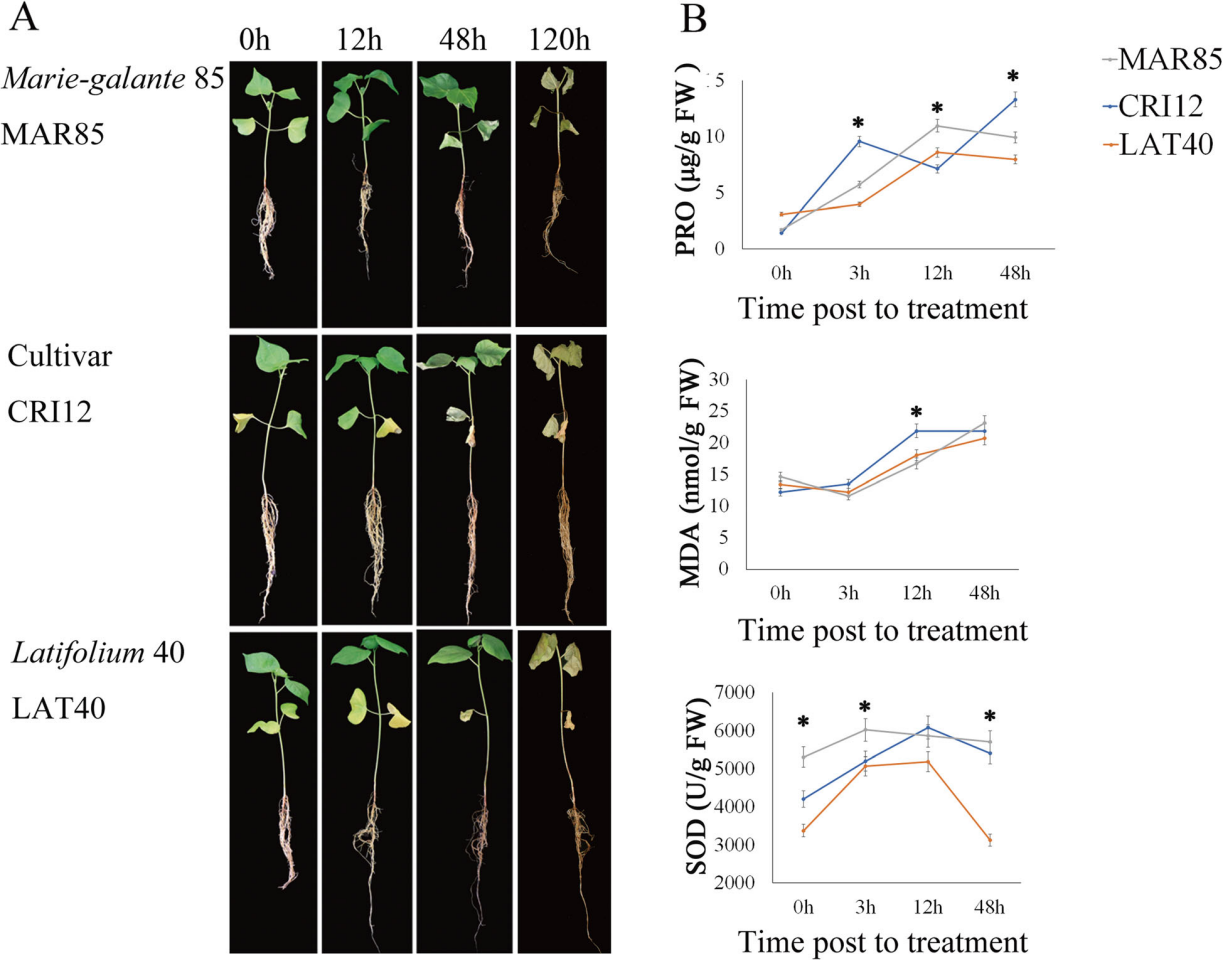
Morphological and physiological characteristics of MAR85, CRI12 and LAT40 after salt-alkali stress treatment. **a** Seedling phenotype at different stages of development in three accessions of upland cotton. **b** The dynamic PRO, MDA and SOD content after salt-alkali stress treatment

The three upland cotton genotypes morphologically are identical and exhibit no significant variations when exposed to any form of stress, thus no consideration of various morphological traits were considered in this study. However, we observed variation on root growth and biomass accumulation, CRI12 and LAT40 had a relatively higher root and overall higher biomass compared to MAR85 (Fig. 1a). Moreover, in carrying out deep analysis on the concentration levels of oxidant and antioxidant enzymes on the leaf tissues of the three cultivars under salt stress conditions, MAR85 and CRI12 showed significantly higher concentrations of proline and superoxide dismutase (SOD) compared to LAT40 (Fig. 1b), an indication that CRI12 and MAR85 were less affected under salt stress compared to LAT40. Furthermore, Malondialdehyde (MDA) concentrations levels within the plants is an indication that the plants are suffering from oxidative stress, being MDA is a byproduct of lipid peroxidation [22]. The results obtained were in agreement to previous findings in which the knockdown of trihelix transcription factor in cotton reduced drought and salt stress tolerance and in turn accelerated the accumulation of various oxidants and MDA levels under drought and salt stress exposure [22].

### Sequencing and transcript identification

The transcription analyses of three accessions of *G. hirsutum* differed significantly in their morphological characteristics as well as their crucial systems-level insights into molecular mechanisms that underlie the alkali-salt stress response [23, 24]. In this study, one upland cotton cultivar (CRI12, China) and two other wild upland cotton cultivars (LAT40, *G. hirsutum* race latifolium40 and MAR85, *G. hirsutum* race mari-garant85) were used for transcription analysis by carrying out RNA-Sequencing on the their tissues when exposed to salinity stress. Two organs were considered, the leaf and root of the three accessions, at four different treatment stages; the samples were coded as Rt_0h, Rt_3h, Rt_12h, Rt_48h for the root samples and leaf samples were coded as Lf_0h, Lf_3h, Lf_12h and Lf_48h, all the samples were replicated three times. The performance of the three upland cotton accessions was categorized into four distinct groups, as normal growth stage (CK0), early alkali-salt stress response stage (SS3), seedlings significantly damaged stage (SS12) and seedling recovered stage (SS48). In order to investigate the transcriptome dynamics throughout alkali-salt stress treatment, total RNA was isolated from the leaf and root of three accessions of *G. hirsutum* in four different treatment stages. In total, 1,106,485,712 numbers of the raw reads were produced from 48 cDNA libraries, after cleaning, 1,097,617,134 (99.19%) clean reads were obtained. The percentage of mapped reads among clean reads in each library ranged from 83.88 to 89.61%, moreover the percentage of clean reads with a Phred quality value of 30% ranged from 94.37 to 97.05%. Furthermore, the clean reads were aligned to the reference genome of *Gossypium hirsutum* (AD_1_), in which 83.06 to 89.73% reads generated from the 48 samples were mapped to reference genome, producing 77.4–82.3% of the uniquely mapped reads to the reference genome*. G. hirsutum* genome. What was more interesting; the uniquely mapped reads were mapped to the reference genome by use of HT-seq (Python package), with higher efficiency and accuracy (Table 1 and https://www.ncbi.nlm.nih.gov/sra/PRJNA531727).

**Table 1:** Quality assessment of raw RNA-seq data

Samples	Sample description	Total Raw Reads	Total Clean reads	Total mapped reads	Unique mapped reads	Read mapped Gene	Detected Gene Number	Clean Reads Q30 (%)
CRI12_0h_Lf	CRI12L_CK0	45,076,438	44,843,338	89.61%	81.76%	67.89%	52,438	95.02
CRI12_3h_Lf	CRI12L_SS3	36,371,236	36,165,990	88.47%	80.32%	66.27%	51,586	95.09
CRI12_12h_Lf	CRI12L_SS12	54,172,122	53,667,540	87.62%	80.42%	64.58%	54,345	95.3
CRI12_48h_Lf	CRI12L_SS48	46,780,058	46,370,366	89.51%	82.27%	67.83%	52,851	95.26
CRI12_0h_Rt	CRI12R_CK0	49,346,508	48,798,312	86.73%	80.54%	65.40%	55,285	95.41
CRI12_3h_Rt	CRI12R_SS3	41,632,004	41,352,494	86.06%	80.25%	64.34%	56,495	95.72
CRI12_12h_Rt	CRI12R_SS12	48,589,958	48,120,064	83.06%	77.40%	63.15%	55,264	95.64
CRI12_48h_Rt	CRI12R_SS48	40,994,510	40,614,906	87.29%	81.28%	66.03%	54,023	95.39
_LAT40_0h_Lf	LAT40L_CK0	47,726,384	47,432,662	88.73%	80.61%	66.47%	53,705	94.73
_LAT40_3h_Lf	LAT40L_SS3	51,429,486	51,097,604	87.88%	80.53%	65.48%	53,530	95.26
LAT40_12h_Lf	LAT40L_SS12	49,696,922	49,450,576	88.86%	81.93%	67.14%	53,544	97.05
LAT40_48h_Lf	LAT40L_SS48	42,032,304	41,587,888	89.07%	81.74%	67.64%	52,073	95.58
LAT40_0h_Rt	LAT40R_CK0	41,946,322	41,582,628	85.71%	79.58%	64.06%	55,615	95.4
LAT40_3h_Rt	LAT40R_SS3	54,179,820	53,844,398	87.90%	82.11%	65.56%	57,263	95.5
LAT40_12h_Rt	LAT40R_SS12	50,560,404	50,200,318	84.77%	78.76%	64.65%	55,536	95.47
LAT40_48h_Rt	LAT40R_SS48	49,685,100	49,157,194	86.71%	80.52%	65.01%	54,298	95.4
MAR85_0h_Lf	MAR85L_CK0	57,496,402	57,163,534	88.71%	80.98%	67.38%	54,338	95.07
MAR85_3h_Lf	MAR85L_SS3	38,567,268	38,288,104	89.73%	82.12%	67.73%	53,318	95.35
MAR85_12h_Lf	MAR85L_SS12	44,010,240	43,575,168	89.29%	82.20%	67.42%	54,173	96.15
MAR85_48h_Lf	MAR85L_SS48	37,316,038	36,970,900	89.06%	81.42%	67.05%	52,613	95.71
MAR85_0h_Rt	MAR85R_CK0	46,322,192	45,881,516	83.88%	77.79%	62.43%	55,098	95.55
MAR85_3h_Rt	MAR85R_SS3	38,207,814	37,865,864	85.65%	79.82%	64.02%	55,040	95.4
MAR85_12h_Rt	MAR85R_SS12	39,974,434	39,617,928	88.30%	82.30%	66.49%	54,798	95.35
MAR85_48h_Rt	MAR85R_SS48	54,371,748	53,967,842	88.31%	82.22%	65.65%	55,054	96.14

*CRI12* Cotton research institute, *G. hirsutum* accession number 12, *MAR86* Marie-galante85, *LAT40* Race latifolium40, *CK0* Normal growth stage, *SS3* Early alkali-salt stress response stage, *SS12* Seedlings significantly damaged stage and SS48: seedling recovered stage (SS48)

### Transcription and expression analysis in various tissues of the three cotton species under salt stress condition

**Fig. 2:**
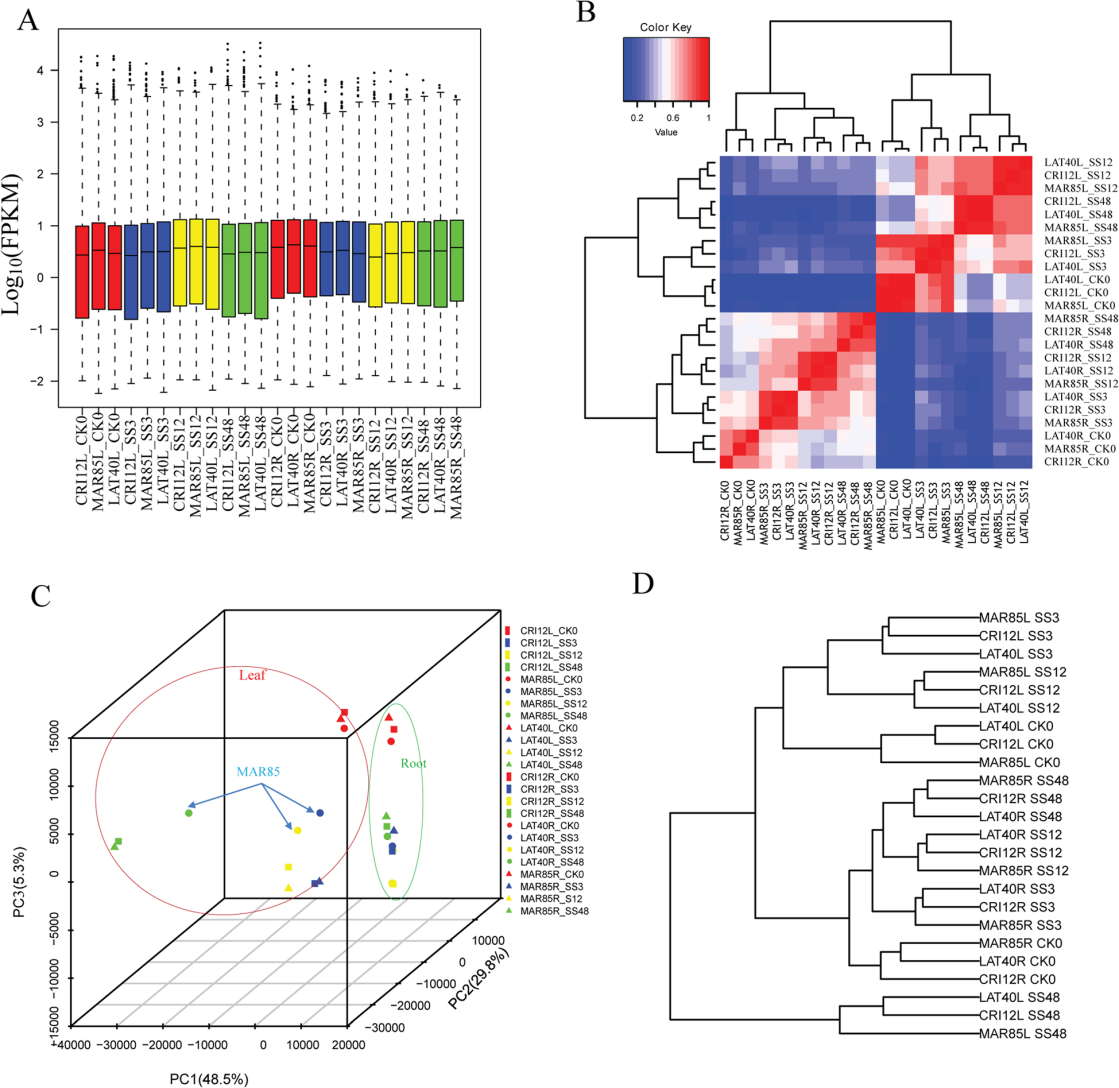
Gene expression analysis of three accessions of *G. hirsutum*. **a** expression level distribution of different samples; **b** Heatmap of sample correlation analysis result base on global transcriptome expression under alkali-salt stress; **c** Plot of Principal component analysis (PCA) base on global transcriptome expression under alkali-salt stress; **d** Clustering of samples base on global transcriptome expression under alkali-salt stress

A total of 64,737 genes were identified, and the number of expressed genes in different samples varied from 51,586 to 57,263 as detected by RNA-sequence data analysis (Fig. 2a). To comprehend the global differences in the transcriptome dynamics during dissimilar treatment stages, the expression distribution analysis, correlation analysis, principal component analysis (PCA) and hierarchical clustering have realistically accomplished based on FPKM values for all the expressed genes in at least one of the 32 tissue samples (Fig. 2b). The leaf samples showed a lower expression level than root, and same treatment tissue/treatment stage of three accessions of *G. hirsutum* shown the similar expression distribution. The Pearson correlation coefficient analysis, among the combined 32 samples illustrated more significant correlation in the same tissue/treatment stage among three accessions of *G. hirsutum* (Fig. 2c). As expected, leaf transcriptome of three contrasting upland cotton races clustered together and showed substantial differences with treatment points. The LAT40 and CRI12 showed a more exact correlation and similar phenotype in the leaf after alkali-salt treatment indicated high similarity in their transcriptional programs. Compared with CK0 stages, SS3, SS12 and SS48 demonstrated closer in the root and leaf of three accessions of upland cotton (Fig. 2d). It suggested a significant difference of transcriptional programs between the standard and alkali-salt stress condition. Overall, tissues/treatment stages exhibited a higher correlation in these analyses that expected to have more similar transcriptomes and functions/activities.

### Differential gene expression after alkali-salt treatment

**Fig. 3:**
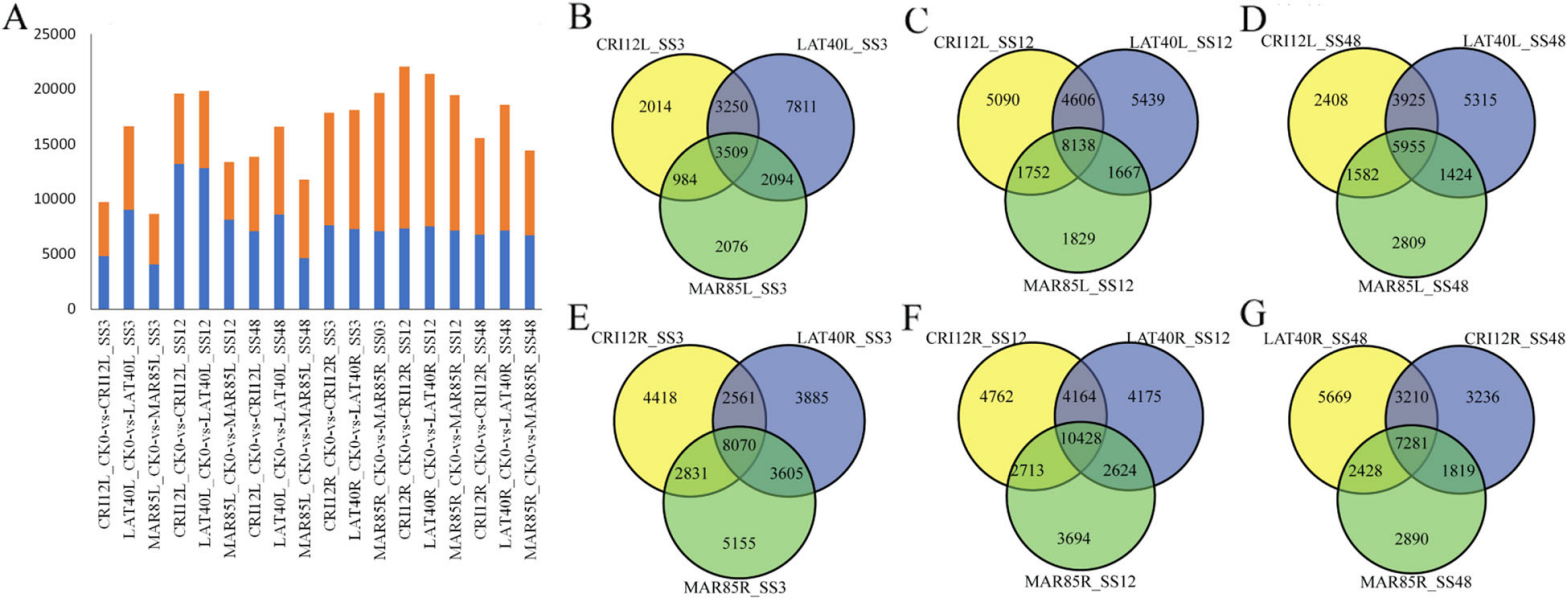
Different expression genes (DEGs) analysis of three accessions of *G. hirsutum*. **a** DEGs number after alkali-salt treatment; **b**-**g** DEGs in different treatment stages in leaf and root (L3 h, L12 h, L48 h, R3h, R12h and R48h), respectively

To investigate the differential expression pattern of the various transcriptional factors in the three upland cotton accessions, two tissues were profiled, the leaf and root tissues under salt stress condition. The total number of the differentially expressed genes (DEGs) varied, ranging from 8663 (MAR85L_CK0 vs. MAR85L_SS12) to 22,068 (CRI12R_CK0 vs. CRI12R_SS12) furthermore, the DEGs were obtained from pairwise comparison (MAR85L: MAR85L_CK0 vs. MAR85L_SS3; MAR85L_CK0 vs. MAR85L_SS12; MAR85L_CK0 vs. MAR85L_SS48; MAR85R, LAT40L/R and CRI12L/R pairwise comparison was similar with MAR85L) by using DEGseq software (Fig. 3a). A greater number of DEGs observed in roots (a total of 41,132 DEGs) than it in leaves (a total of 35,724 DEGs) which indicated that the roots could be the main tissue affected by salt stress and thus have a more dynamic and complex gene regulation to reduce the toxicity of salts to the root cells. The results obtained are in agreement to previous findings in gene expression pattern tends to be tissue specific Magwanga et al. [25], found that high number of the *LEA* genes were highly upregulated in the leaf tissues compared to stem and root tissues under drought stress condition. Compared with SS3 (21,738 and 30,525, in leaves and roots, respectively) and SS48 (23,418 and 26,533, in leaves and roots, respectively) stages, the SS12 stage (28,521 and 32,560, in leaves and roots, respectively) exhibited the highest number of DEGs in root and leaf, indicating SS12 stage was more activated for response to alkali-salt stress. Interestingly, MAR85 showed the lowest number of the DEGs in all the stages compared to LAT40 (highest number of DEGs) and CRI12. It indicated that the inconsistent responses to alkali-salt stress of three accessions of upland cotton. Additionally, 3509 (SS3 VS. CK0) 8138 (SS12 VS. CK0), and 5955 (SS48 VS. CK0) common DEGs were identified at each stage subsequently alkali-salt treatment in the leaves of three accessions of upland cotton. Then, 8870 (SS3 VS. CK0), (Fig. 3e), 10,428 (SS12 VS. CK0), and 7281 (SS48 VS. CK0), DEGs were identified in the roots (Fig. 3b). The variable number of differentially expressed genes suggested that each tissue/alkali-salt treatment stage retained their own independent developmental programs. Alternatively, the transcriptional complexity may merely reflect the intricacy of the captured seed stages, which contained more than one cell type.

### Functional annotation of DEGs sets in the three accessions of upland cotton at different tissues (leaf and root)

A total of 22,359 DEGs (11,818 and 15,674 in leaf and root, respectively) was used for functional enrichment and weighted co-expression network analysis (WGCNA). The cluster analysis, consequence of the 27,492 DEGs was similar to the cluster analysis of all the genes. This empirical observation supported those 22,359 DEGs as obtained from the leaf and root tissues in the three different cotton accessions could precisely illustrate the variations in the samples analyzed. The common DEGs of each tissue/stage in three accessions of upland cotton were further used for carrying out the gene ontology (GO) enrichment and Kyoto encyclopedia of genes and genomes (KEGG) enrichment analysis. A total of 17,985 genes were annotated, with 7836 and 10,149 being found to be differentially expressed in the leaf and roots, respectively. Among the 7836 annotated DEGs, 105 GO terms were significantly enriched at *P*-value ≤0.05, FDR ≤ 0.05 (Additional file 6: Table S2). In roots, more of the DEGs were annotated, however, only 96 GO terms were found to be significantly enriched (Additional file 7: Table S3). Moreover, 54 GO terms were commonly enriched between the leaf and root tissues, including oxidation-reduction process (GO: 0055114), carbohydrate metabolic process (GO: 0005975), oxidoreductase activity (GO: 0016491), protein serine/threonine kinase activity (GO: 0004713) and transcription factor activity (GO: 0003700), which were known to be involved abiotic stress response.

Tissue-specific enrichment GO terms were observed where the DEGs obtained from the leaf were significantly enriched in photosynthesis (GO: 0015979) and photosynthesis-related GO terms (GO: 0019684, GO: 0009765). Moreover, all the 4163 DEGs (2426 and 2667 DEGs in leaves and roots, respectively) were enriched by using the KEGG database. The DEGs of the leaf and root were significantly enriched in 30 and 34 KEGG terms, respectively, with the Q value of *≤0.0001*, and gene number for each analyzed term set at ≥3 (Additional file 8: Table S4 and Additional file 9: Table S5). Plant hormone signal transduction (ko04075) and the biosynthesis of secondary metabolites (ko01110) were the most common KEGG pathways detected (Additional file 2: Figure S1 and S2), and the two pathways have been previously found to be significantly involved in abiotic stress response [26–29]. Moreover, the leaf-specifically enriched KEGG terms detected were those related to the photosynthetic apparatus of the plant leaf, such as Photosynthesis (ko00195) and Photosynthesis - antenna proteins (ko00196). Even though alkali-salt stress response do involved in a more complex pathways, signal transduction and oxidation-reduction processes, but the DEGS detected could be playing a core role in the leaf and root tissues of cotton under salt stress conditions. Apart from the DEGs, we analyzed the various plant transcription factors with putative function in enhancing salt stress tolerance among the three cotton cultivars used. A total of 2080 TFs were identified, in which 991 TFs were detected among the DEGs in the leaf tissues while 1577 TFs were from the DEGs analyzed from the root tissues roots (Additional file 10: Table S6). The results obtained for the TFs correlated positively with the distribution of the DEGs in which more were found to be expressed in the roots as opposed to the leaf tissues, an indication that the roots could be the primary plant tissue, which is highly affected under salt stress conditions. In all the TFs identified, 53 different gene families were found to be linked to the TFs such as ZF-HD, SRS, S1Fa-like, SAP and NF-X1 TFs families were identified for the DEGs for the root tissues while YABBY TF family, was detected for the DEGs found for the leaf tissues.

### Co-expression network and module construction

To investigate the gene regulatory network of alkali-salt stress response in upland cotton, we identified co-expressed gene sets via weighted gene co-expression network analysis (WGCNA) which can be used to find networks (modules) of highly correlated genes [30]. In the present study, 22,359 DEGs were used for WGCNA. In order to ensure a scale-free network, the power of *β = 12* (scale free R2 = 0.8740) was accurately selected by determination of soft-thresholding power (Additional file 3: Figure S3A/B) and evaluation of scale free topology analysis (Additional file 3: Figure S3C/D). Representing interaction among genes with similar expression profiles, several networks that were referred to as co-expression modules, were identified. A total of 20 modules was identified via the Dynamic Tree Cut method (core parameter: MEDissThres = 0.25), varying from 135 genes in the mistyrose module to 4793 genes in the darkmagenta module. Among 20 modules, 17 genes were not useful, which displayed in the grey module, could not be selected by any other modules (Additional file 4: Fig. 4a-b). A thousand genes were randomly selected for visualization of gene networks. Genes in the same module shown a higher topological overlap (Additional file 4: Figure S4), indicating networks (modules) which were constructed by WGCNA have biological useful functions.

**Fig. 4:**
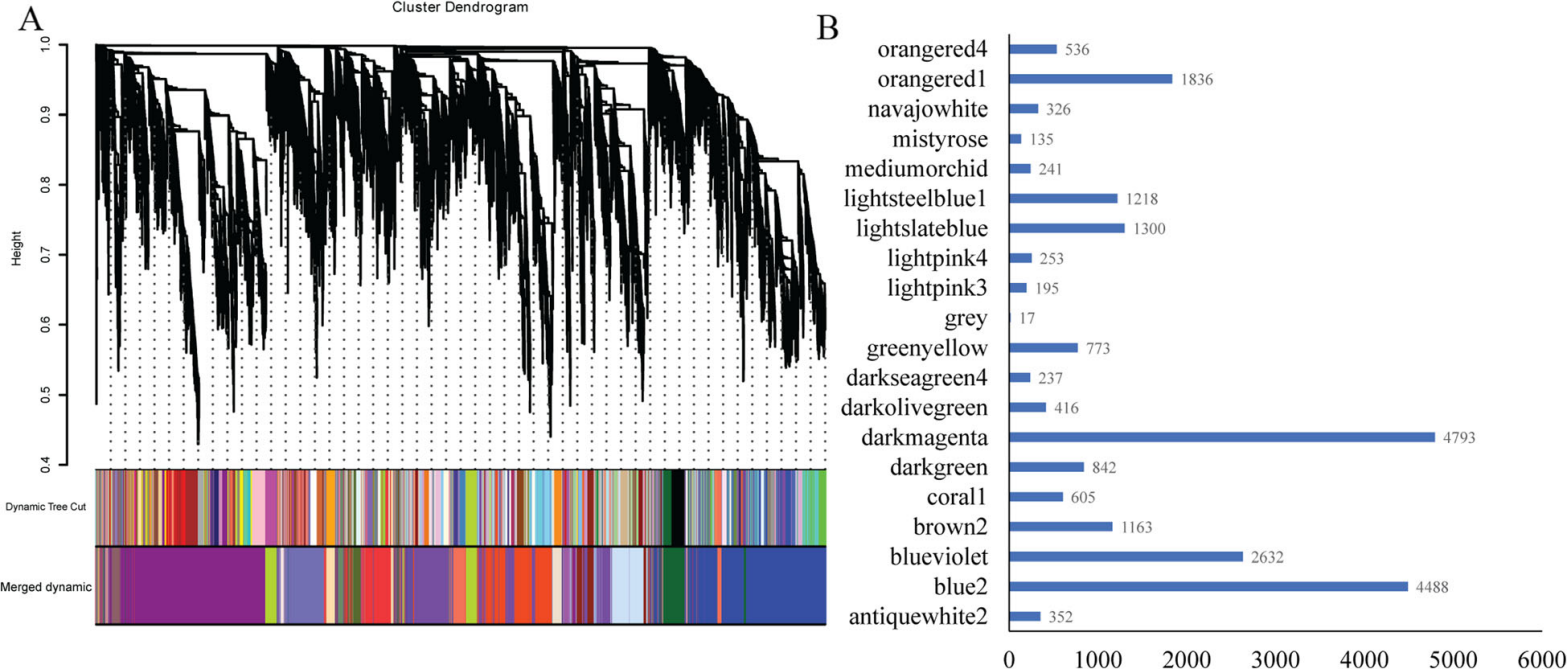
Co-expression network construction of three accessions of upland cotton. **a** Clustering dendrogram of 22,359 different expressed genes, with dissimilarity based on topological overlap. The color rows provide a simple visual comparison of module assignments (branch cuttings) based on the dynamic hybrid branch cutting method. **b** Gene number of each networks/modules

### Identification and visualization of tissue/stage-specific modules in leaf and root of upland cotton

**Fig. 5:**
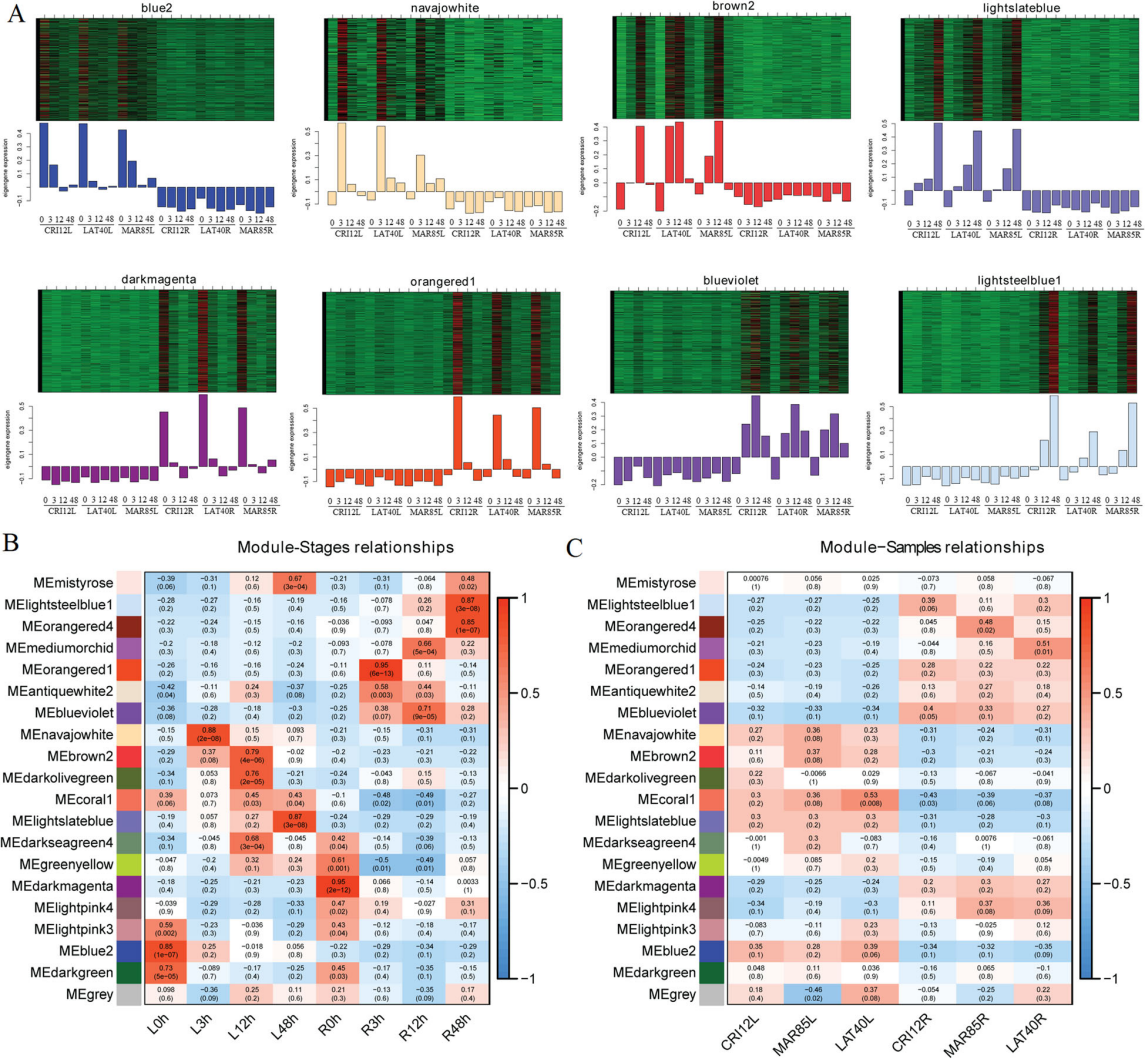
Visualization of gene expression levels and eigengene values of significant modules. **a** Clustering heatmap and bar plot represent gene expression levels of each module. In the heatmaps, the colors range from green to red, indicating low to high expression levels, respectively. **b** Heatmap of correlations between module and different treatment stages in leaf and root. **c** Heatmap of correlation between modules and three accessions of upland cotton in leaf and root. The colors ranging from blue through white to red indicate low through intermediate to high correlations, respectively. ME, the first principal component of the standardized expression profiles of a given module

A summary profile (eigengene) for each module obtained by WGCNA. For example, clustering heat map and eigengene bar plot of modules that highly correlated with traits, which were shown in (Fig. 5a), it represented the gene expression levels of each module. In order to determine the correlation between module and trait, we associated eigengenes with alkali-salt treatment stages and upland cotton races via Pearson correlation coefficient analysis, respectively. Interestingly, none of the module significantly associated with three upland cotton races (*r ≥ 0.60*, *p ≤ 0.01*); however, 11 co-expression modules showed a relatively higher correlation (*r ≥ 0.60*) with SS3, SS12, SS48 stages in leaf and root of upland cotton (Fig. 5b-c). It suggested that the co-expression networks were significantly different at different stages. On the contrary, none of the co-expression networks were found to be associated with three accessions of upland cotton, indicating that a particular regulatory network of alkali-salt stress response in different upland cotton races. SS12 and SS48 stages were correlated with more than one module. SS12 stage was correlated with the brown 2 (Gene number: 1163, *r = 0.79*), dark olive green (Gene number: 416, *r = 0.76*) and dark sea green 4 (Gene number: 237, *r = 0.68*) modules in leaf, and correlated with medium orchid (Gene number: 241, *r = 0.66*) and blue violet (Gene number: 2632, *r = 0.71*) modules in the root, respectively. SS48 stage was associated with misty rose (Gene number: 135, *r = 0.67*) and light slate blue (Gene number: 1300, *r = 0.87*) modules in leaf, and associated with light steel blue1 (Gene number: 1218, 0.87) and orange red 4 (Gene number: 536, *r = 0.85*) modules in the root, respectively. While, only one module was correlated with SS3 stage (navajo white (Gene number: 326, *r = 0.88*) moduleSS3 stage in leaf; orange red1 (Gene number: 1836, *r = 0.95*) module-SS3 stage in root). Interestingly, the highly and significant correlated modules were all positively correlated with different stages, indicated that different expression genes in different tissues/stages were predominantly upregulated to response to alkali-salt stress.

### GO enrichment analysis

We performed GO enrichment analysis of genes in tissue/stage-specific modules. The enriched GO terms are presented in (Additional file 12: Table S8). GO analysis categorizes the genes into three possible groups, namely cellular component (CC), molecular functions (MF) and biological process (BP). The molecular function detected for the DEGs were signal transducer activity (GO:0004871) and phosphorelay response regulator activity (GO:0000156) which were significantly enriched at the SS3 stage, protein tyrosine kinase activity (GO:0004713), protein kinase activity (GO:0004672) and protein serine/threonine kinase activity (GO:0004674) were top three highly enriched at the SS12 stage in leaf; whereas regulatory region nucleic acid binding (GO:0001067), transcription regulatory region DNA binding (GO:0044212), regulatory region DNA binding (GO:0000975) were top three significantly enriched at the SS48 stage (*P < 0.001*, *FDR < 0.05*) in leaf. In the GO enrichment analysis for the biological process (BP), no significant pathway was identified at the SS3 stages in leaf; protein phosphorylation (GO:0006468), macromolecule modification (GO:0043412) and protein modification process (GO:0036211) were the top three pathways at the SS12 stage in leaf; whereas single-organism metabolic process (GO:0044710), carbohydrate metabolic process (GO:0005975) and oxidation-reduction process (GO:0055114) were the top three pathways at the SS48 stage in leaf. For GO analysis of molecular function, nucleic acid binding transcription factor activity (GO:0001071), transcription factor activity, sequence-specific DNA binding (GO:0003700) and oxidoreductase activity acting on paired donors, with incorporation or reduction of molecular oxygen (GO:0016705) were top three significantly enriched at the SS3 stage in root; Additionally, UDP-N-acetylmuramate dehydrogenase activity (GO:0008762), chromatin binding (GO:0003682), macromolecular complex binding (GO:0044877) were top three highly enriched at the SS12 stage in root; whereas oxidoreductase activity (GO:0016491), Acyl-CoA dehydrogenase activity (GO:0003995) and catalytic activity (GO:0003824) were top three significantly enriched at the SS48 stage in root. Interestingly, oxidoreductase activity was found at different stages of differentiation. In the GO enrichment analysis of biological process, regulation of RNA biosynthetic process (GO:2001141), regulation of transcription, DNA-templated (GO:0006355), regulation of nucleic acid-templated transcription (GO:1903506) were top three significantly enriched GO terms in SS3 stage in root; regulation of RNA metabolic process (GO:0051252), regulation of RNA biosynthetic process GO:2001141and regulation of transcription, DNA-templated (GO:0006355) were the top three pathways at the SS12 stage in root. Similarly with the SS12 stage in leaf, single-organism metabolic process (GO: 0044710), single-organism process (GO: 0044699) and oxidation-reduction process (GO: 0055114) were the top three pathways at the SS48 stage in root. Overall, signal transduction was performed at SS3 and SS12 stages and regulates transcription was performed at SS48 stage in leaf. Although it is complicated to regulate alkali-salt stress in root, regulation of oxidation-reduction was performed in all stages. The results obtained were in agreement to previous findings in which similar GO terms have been identified for various stress responsive genes such as *LEA* genes [25], *MATE* genes [31] among others.

### Identification of central and highly connected genes

**Fig. 6:**
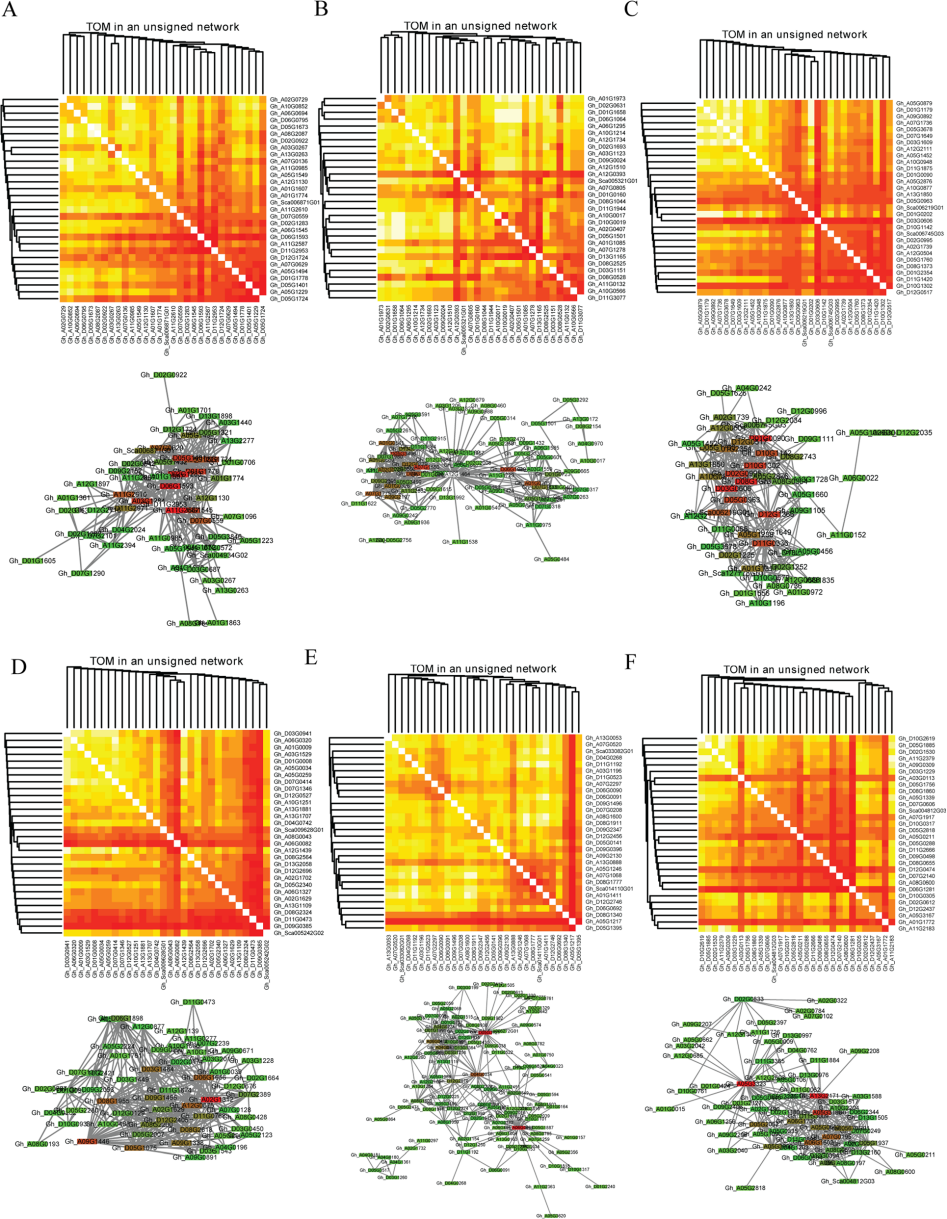
Visualization of connections of genes in various modules by heatmap and Cytoscape 3.3.0. In the top part of the A-F picture, heatmap plots depicting the relationships among the 30 most significant genes identified by network screening. Each column and row of the heat map corresponds to a single gene; light colors mean topological overlaps; progressively darker colors correspond to higher topological overlaps. In the lower part of the A-F picture, connections of 30 hub genes which were selected from top 150 highly connected genes and the top 300 connections of highly connected genes of higher correlation modules. Plots A-F depicts the *hub* gene network of L3 h, L12 h, L48 h, R3h, R12h and R48h, respectively

To identify hub genes under the salt-alkali stress, two different methods were used for screening genes. Each gene possibility weight (*p*. weight) value was obtained by the network screening function of the WGCNA package based on gene significance (GS), modular membership (MM), and lower *p*. The weight value indicated that the gene was a higher correlation with traits (treatment stages). The top 30 *hub* genes were identified based on p. Weight. In the second method, the top 150 and top 300 highly connected genes for higher correlation modules of different stages were selected for analysis based on the topological overlap matrix (TOM) of all differential expression genes. In addition, the top 30 genes that were central and highly connected were visualized using the Cytoscape 3.3.0 software (Additional file 11: Table S7, Fig. 6a-b). In total, 180 *hub* genes of higher correlation with different treatment stages were found and 180 higher connectivity genes were also selected in six different stages, in which 39 common genes were identified by two different methods that indicated the core role of these genes in response to alkali-salt stress. Interestingly, 19 and 18 common genes were obtained in SS3L and SS48L stages, but none of the common genes was found in other stages. Transmembrane proteins (Pfam: DUF3082), *Gh_D11G2953* and *Gh_A11G2587*, were the most correlated genes with other genes and at SS3L stages under alkali-salt stress. Galactosyltransferase family protein (*Gh_D05G1401* and *Gh_A05G1229*) and lysine decarboxylase family protein (*Gh_D05G1724* and *Gh_A03G0267*) were also observed an indication that these genes were significantly important factors at the SS3L stage in relation to salt stress tolerance.

Three different starch branching enzymes (*Gh_A02G1739*, *Gh_D02G0995* and *Gh_Sca006745G03*) and two RING/U-box superfamily proteins (*Gh_D05G0963* and *Gh_D07G1649*) were identified which could be playing an important role in SS48L stages. Homolog gene pairs were identified at the same time by WGCNA, suggested their potential key roles in the regulation of alkali-salt stress in cotton. A total of 35 TFs was found among *hub* genes, out of 26 TFs showed higher connectivity to various DEGs. Moreover, among the 35 TFs, 4 of the common genes had a higher correlation with other genes and treatment stages under alkali-salt stress, were members of the double B-box zinc finger (DBB, *Gh_A10G0877* and *Gh_A11G2610*) and nuclear factor YA (NF-YA, *Gh_D03G0606* and *Gh_Sca006219G01*) TF family. Overall, 321 *hub* genes were identified, including 35 transcription factors.

### Validation of the hub genes by RT-qPCR

**Fig. 7:**
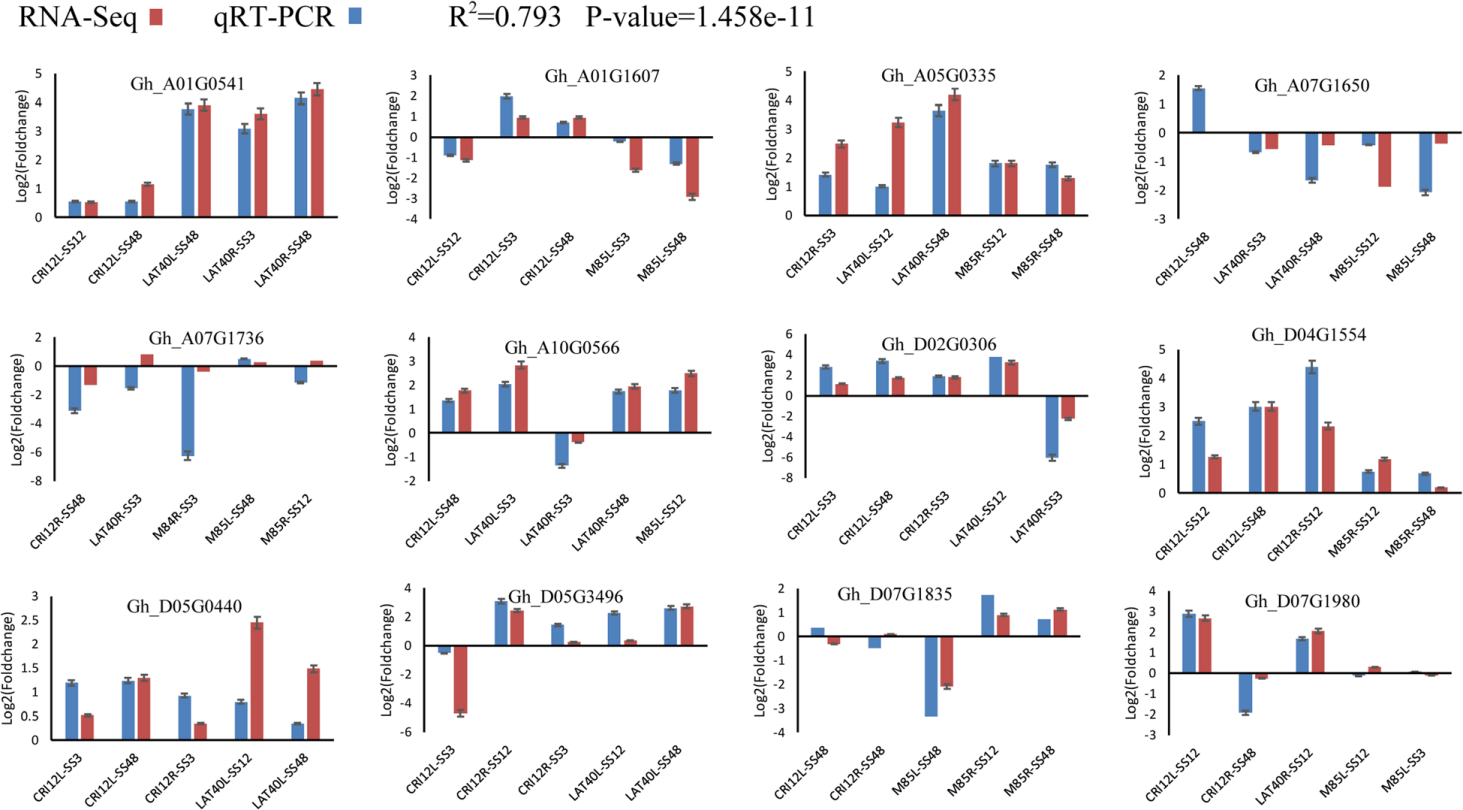
Validation of hub genes by RT-qPCR

In order to detect the expression level and function of the *hub* gene, 12 genes were selected and their expression analyses. In each of the selected genes were randomly examined in five samples by RT-qPCR. The gene primer information of 12 *hub* genes was designed and details are contained in (Additional file 5: Table S1, Additional file 6: Table S2, Additional file 7: Table S3, Additional file 8: Table S4, Additional file 9: Table S5, Additional file 10: Table S6, Additional file 11: Table S7, Additional file 12: Table S8). We found that the gene expression level data from RT-qPCR were significantly highly correlated (cor = 0.7397, *p*-value = 1.458e-11) with the data from RNA-seq (Fig. 7a). This result proved the RNA-seq data were reliable in this research. Moreover, compared with the expression level of control seedlings, the hub genes were differentially expressed under salt stress conditions, which suggested that the hub genes identified by WGCNA had a putative role in salt-alkali stress response.

### Enhanced salt sensitivity in *GhSOS3* and *GhCBL10* virus-induced gene silencing (VIGS) seedlings

**Fig. 8:**
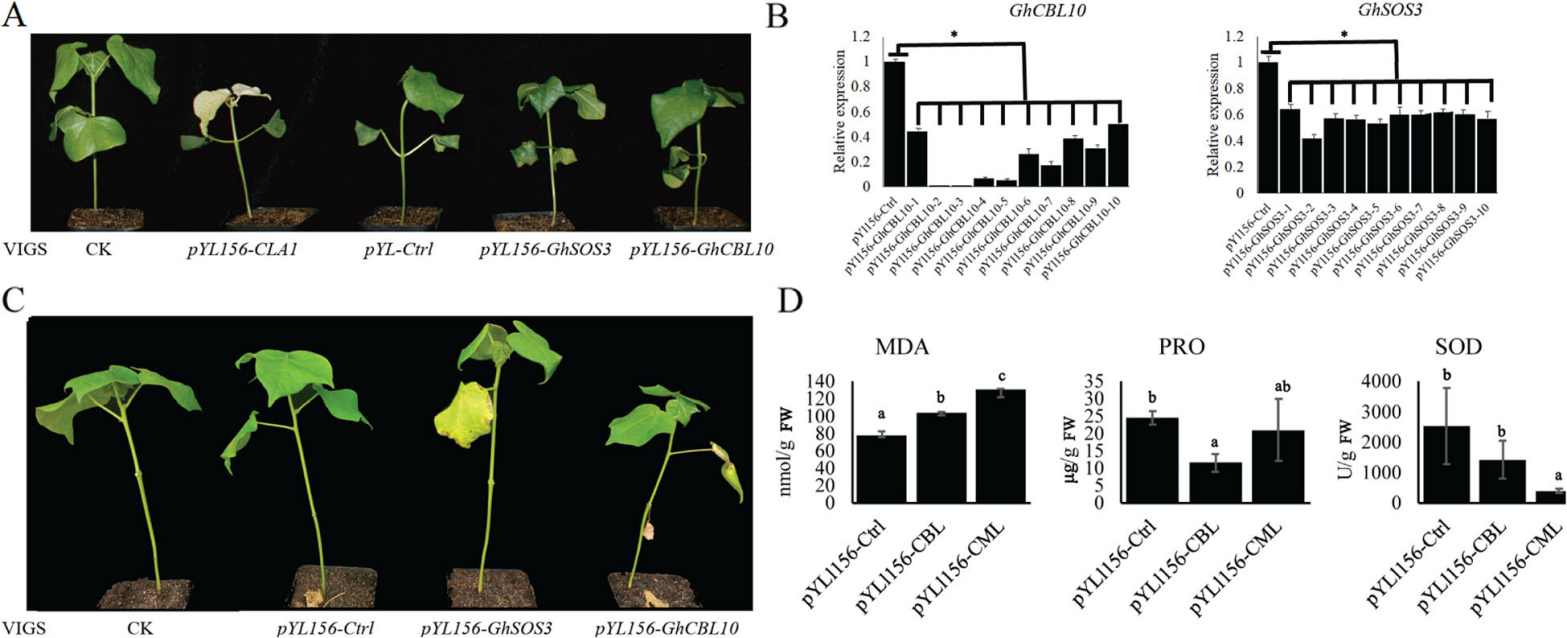
Phenotype evaluation, expression analysis and biochemical Assays on the VIGS and wild type plants under Alkali-salt stress. **a** The phenotype of normal (CK), pYL156-Ctrl, pYL156-PDS, *pYL156-GhSOS3* and *pYL156-GhCBL10* seedlings of MAR85. **b** The phenotype of normal (CK), pYL156-Ctrl, pYL156-PDS, *pYL156-GhSOS3* and *pYL156-GhCBL10* seedlings of MAR85 after 20 days post to salt-alkali stress treatment. **c** The expression level of *GhCBL10* and *GhSOS3* genes on the VIGS, WT and positively controlled plants under normal condition (**d**) PRO, MDA and SOD content in normal (CK), *pYL156-Ctrl, GhSOS3* and *pYL156-GhCBL10* seedlings of MAR85 after salt-alkali stress treatment

To further investigate the functions of hub genes, *GhSOS3* and *GhCBL10*, the VIGS pYL156-GhPDS, pYL156-Ctrl, *pYL156-GhSOS3* and *pYL156-GhCBL10* plants were observed under the salt-alkali stress. Albino leaves were observed in pYL156-PDS inoculated seedlings after 7 days of inoculation. Compared with infected seedlings, we found that control seedlings had rapid growth after 20 days of inoculation. Moreover, no differences were noted between infected seedlings (Fig. 8a). The expression levels of *GhSOS3* and *GhCBL10* were checked by RT-qPCR Compared with pYL156-Ctrl seedlings, expression levels of *GhSOS3* and *GhCBL10* were down-regulated in the corresponding gene silencing seedlings after 20 days of inoculation (Fig. 8b). The leave of *pYL156-GhSOS3* and *pYL156-GhCBL10* seedlings withered and wilted compared with the control and the pYL156-Ctrl seedlings after 20 days of salt-alkali stress treatment (Fig. 8c). Additionally, the PRO and SOD content was lower in *pYL156-GhSOS3* and *pYL156-GhCBL10* seedlings compared with control and pYL156-Ctrl seedlings after 20 days of salt-alkali stress treatment. On the contrary, the MDA content was higher in *pYL156-GhSOS3* and *pYL156-GhCBL10* seedlings compared with control and pYL156-Ctrl seedlings (Fig. 8d). This result suggested *pYL156-GhSOS3* and *pYL156-GhCBL10* seedlings sensitivity was enhanced.

## Discussion

### Plant response to abiotic stress condition

The molecular mechanisms of abiotic stresses such like soil salinity, drought, and extreme temperatures response are very complicated and imperfectly understood in upland cotton, although many researchers go through much effort on it [32]. In recent years, next generation sequencing technology (NGS) was used to investigate the mechanism of abiotic stress response in plant [33].

Soil salt-alkalization remain a critical limiting factor for crop production in many regions [34]. However, most previous studies have merely pinpointed on salt stress in plants. As with salt stress response [35], roots and leaves are two most primary important tissues in the response to alkali-salt stress in plants. In this empirical study, the roots and leaves of three accessions of upland cotton were used for analysis under alkali-salt stress condition. We implemented RNA-seq approach to detect the transcriptome dynamics in three accessions at different stages of alkali-salt stress treatment, and investigated the molecular mechanism regulation of alkali-salt stress response.

### Transcription and expression analysis in various tissues of the three cotton species under salt stress condition

A total of 70,478 predicted-coding genes consist in the allotetraploid cotton *G. hirsutum* genome (TM-1) [36], 91.85% (64,737) of the upland cotton genes were found to be expressed in at least one sample of three accessions of upland cottons. The distribution of expression levels across different samples revealed significant similarity among three different upland cotton materials at the same tissues/treatment stages. It is also suggested the reproducibility of expression data under alkali-salt stress and the similar models of alkali-salt response at the same treatment tissue/stage. Each tissue/stage was clearly distinguished in the PCA plot, a heatmap of correlation coefficient and cluster graph, suggesting that significant changes in gene expression typically occur from one stage to another. The leaf and root have different expression model under alkali-salt stress, demonstrating the different mechanism of the root and leaf in response to alkali-salt stress [37]. A distinct relationship between the expression level of normal growth condition and expression level under the salt-alkali stress, indicating there is a regulation of expression mechanism at the transcriptome level. Furthermore, we found that each tissue/stage of three different upland cotton materials showed the clearly close relationship. This result suggested there is the similar regulation model at the same tissue/stage, although three upland races obviously have the different phenotypic and genetic backgrounds. Overall, the global transcriptome of *Gossypium hirsutum* races reveal tissue/stages-specific mechanism under salt-alkali stress.

Among three accessions of upland cotton, 3509, 8138 and 5955 common DEGs were found at SS3, SS12 and SS48 stages in leaf, respectively. Moreover, 8070, 10,428 and 7281 common DEGs were found at SS3, SS12 and SS48 stages in root, respectively. More DEGs indicating complex mechanism, which was initiated in the roots of the upland cotton races. What is more, more DEGs were at SS12 stage than SS3 and SS48 stages, indicating regulation of salt-alkali stress response was more active at SS12 stage. Twenty two thousand three and fifty-nine (31.72%) representative differently expressed genes were identified and used for gene co-expression network (GCN) construction. In our study, we constructed a GCN of upland cotton in response to salt-alkali stress and identify tissues/stages-related modules by transcriptome data using weighted gene co-expression network analysis (WGCNA). WGCNA which is a systems biology method for describing the correlation patterns among genes across microarray samples was used for finding modules/network of highly correlated genes [18]. Recently, WGCNA was wildly used for biological and medicinal study [38, 39].

In our analysis, nineteen modules/networks were identified by WGCNA. Tissue/stage and three accessions of upland cotton have been used as traits to associate with modules, respectively. Highly correlated modules were identified between tissue/stages and modules, but none module has been found between different upland races and modules. Interestingly, in the previous analysis, we found expression model of all samples were divided six tissue/stage-specific clusters. Consistent results proved regulation of salt-alkali stress response significantly is specific to tissue/stage. What is more, 11 module co-expression networks/modules, which significantly associated with different tissue/stages, were identified by WGCNA.

### Co-expression network and module construction

In leaf, the SS3 stage was positively correlated with navajowhitemodule; SS12 stage was significantly correlated with the brown2, darkolivegreen and darkseagreen4 modules; SS48 stage was associated with mistyrose and lightslateblue modules. Based on the result of GO enrichment of modules genes, SS3 stage was involved in “phosphorelay response regulator activity” and “signal transducer activity”; SS12 stage was involved in “protein kinase activity”, “phosphotransferase activity”, “protein serine/threonine kinase activity” and so on; SS48 stage was involved in “single-organism metabolic process”, “carbohydrate metabolic process”, “oxidation-reduction process” and so on. Among *hub* genes in leaf, 17 transcription factors were identified, including WRKY, DBB, MYB, C3H, NF-YA and ERF. It has been demonstrated that CCCH-type zinc finger protein from cotton enhances salt stress tolerance [40]. As large transcription factor family, some *WRKY*, *bHLH* and *MYB*-coding genes involved in salt stress response [41]. In addition, early responsive to dehydration stress protein (*ERD*)-coding gene, calcium-binding EF-hand family protein (*SOS3*)-coding gene and most of serine/threonine-protein kinase were observed involved salt-alkali stress response. Overall, the result showed that genes at SS3 and SS12 stage were involved in salt signal transduction and genes at SS48 was involved in oxidation-reduction and carbohydrate metabolic in leaf.

In root, the SS3 stage was correlated with orangered1; SS12 stage and correlated with medium-orchid and blue-violet modules; SS48 stage associated with lightsteelblue1 and orangered4 modules. Reactive oxygen species (ROS) control many different biological processes in plants, including abiotic stress response [42]. In our study, based on the result of GO enrichment of modules genes, genes at all the stages were involved in “oxidoreductase activity” and “oxidation-reduction process” in root. The higher tolerance cotton cultivars and better antioxidant defense capacity [43]. This result indicated that oxidation-reduction process plays an important role in the salt-alkali stress response of cotton root.

### Identification of central and highly connected genes

*Hub* genes were considered a good representation of a module in network biology [44]. In our study, 321 *hub* genes were selected as the key members to regulate of salt-alkali stress response. It is complicated that regulation of alkali-salt stress response and many of biological processes were involved. The secondary effects of salt stress include oxidative stress and damage to cellular components [45]. In previous study, salt overly sensitive (SOS) signal network-mediated oxidation balance regulates salt stress response in cotton [6]. Reactive oxygen species (ROS) as signaling molecules to control various processes including pathogen defense, programmed cell death, and abiotic and biotic stress response [46]. As with salt stress, signal transduction and oxidation-reduction process were involved alkali-salt response in both leaf and root. Further, 24 (6.67%) *hub* genes were enriched in oxidation-reduction process. Abiotic stress signaling in plants evolved from energy sensing [45] and protein kinases involved in abiotic stress signaling pathways. Twenty-eight (7.78%) protein kinases-coding genes were found in hub genes, including 16 genes, which were enriched protein serine/threonine kinase activity (GO: 0004674). It means those two processes play a core role in salt-alkali stress response in cotton. SOS signal pathway, the abiotic stress signaling pathway, were established in plants [9], including several core components, *SOS1/2/3*. Calcium-binding EF-hand protein (SOS3) [10], interacts with and activates *SOS2*, a serine/threonine protein kinase [47]. Among *hub* genes, *SOS3* paralogs, calcineurin B-like protein 10 (CBL10), performs an equivalent role as SOS3 [11]. SOS3 and CBL act preferentially in roots and shoot under salt stress in *Arabidopsis thaliana*, respectively. However, in our study, *SOS3/Gh_A01G1607*, were identified in leaf at SS3 stage and *CBL10*, *Gh_D05G0440* and *Gh_A05G0335*, were identified in root at SS48 stage. SOS2, plant-specific serine/threonine-protein kinase, represents a large protein kinases family. These proteins are generally referred to as sucrose non-fermenting-1-related protein kinases (SnRKs) whose have the similar catalytic domain with the yeast sucrose nonfermenting 1 (SNF1) and mammalian AMP-activated protein kinase (AMPK), including 20 SnRK2 in upland cotton [48]. Among hub genes, *Gh_A07G1736*, SNF1-related protein kinase regulatory subunit beta-2 (KINB2), who referred to as regulatory subunit of SnRKs complex [49], may play a role in a signal transduction cascade regulating alkali-salt stress response via interacting with *SOS3* or *CBL*. Overly, under salt-alkali stress, there may be a *GhSOS3/GhCBL10*-*GhSOS2* network in upland cotton that was similar to SOS signal pathway in *Arabidopsis thaliana*. Although leaf and root have different regulation mechanism of salt-alkali stress response in upland cotton, signal transduction and oxidation-reduction process were enriched in both leaf and root, indicating the important role of those two processes in upland cotton.

## Conclusions

Next generation sequencing technology conveniently provides a potential tool for promptly investigating the complex mechanism of abiotic stress response in the plant. RNA-seq data, generated from three accessions of upland cotton with contrasting phenotype in this study, present a resource to study salt-alkali stress response. A total of 22,359 (31.72%) differentially expressed genes were reliably identified in our investigation. Moreover, gene sets of tissue/stage-specific modules were precisely identified via WGCNA and determined their GO enrichment terms. We also defined, the co-expressed gene sets with significant correlation with different tissues/stages. Gene expression analysis, different expressed gene analysis and WGCNA indicated tissue/stage-specific models of specific regulation of salt-alkali stress in upland cotton. The GO enrichment analysis suggested signal transduction process involved in salt-alkali stress response at SS3 and SS12 stages in leaf; carbohydrate metabolic process and oxidation-reduction process involved in salt-alkali stress response at SS48 stages in leaf; the oxidation-reduction process involved in salt-alkali stress response at all three phases in the root. Our result of hub gene analysis typically revealed that the *GhSOS3/GhCBL10-SOS2* network plays a core role in specific regulation of salt-alkali stress in upland cotton. Salt-alkali sensitivity of *GhSOS3* and *GhCBL10* Virus-induced Gene Silencing (VIGS) seedlings were enhanced. It further proved *GhSOS3* and *GhCBL10* involved in salt-alkali stress. Overall, present studies suggest that global transcriptome and deduced co-expression network analysis can facilitate identification of the most promising candidate genes and establish their precise role in alkali-salt stress response in upland cotton.

## Methods

### Plant materials and salt stress treatment

Two accessions of MAR85 and LAT40, presenting *G. hirsutum* race marie-galante85 (TX-1771) and race latifolium40 (TX-70), respectively, and one *G. hirsutum* cultivar CRI12 were used in this study. The three cotton species are tetraploid, and are varied in terms of their performance. MAR85 is highly tolerant to drought and salt-alkali stresses [50], CRI12 is relatively tolerant to various stress factors [51, 52], while LAT40 is highly susceptible to both drought and salt stress factors [53]. There are seven races (also called as semi-wild cotton) in *G. hirsutum* and they are originally distributed in Mexico. Here the accessions MAR85 and LAT40, distributed in Guadeloupe and Guatemala, respectively, were introduced from USDA-ARS Southern Agricultural Research Center in College Station, Texas, USA and perennially preserved in the National Wild Cotton Nursery, which locates in Sanya, Hainan, China and is supervised by Institute of Cotton Research, Chinese Academy of Agricultural Sciences (ICR-CAAS). The cultivar CRI12 was developed by ICR-CAAS and commercially used as an only cultivar for the most years, largest acres, as well as planted simultaneously in the three cotton regions in China from 80S to 90S of the last century and won the first class prize of National Technical Innovation awarded by Chinese government in 1990.

The seeds of MAR85, LAT40 and CRI12 were first germinated at 28 °C in a 16 h light/8 h dark cycle. Then, seedlings were planted in the normal solution for three weeks. The similar growth seedlings were selected for salt-alkali stress. According to the ion components in the saline and alkaline land of Xinjiang, China, salt-alkali stress solution composition was designed via several experiments of seedling growth [54]. The composition of the salt-alkali solution was formulated by combining CaCl_2_ (0.0970 molL^− 1^), NaHCO_3_ (0.0086 molL^− 1^), Na_2_SO_4_ (0.1411molL^− 1^), K_2_SO_4_ (0.0097 molL^− 1^), MgSO_4_.7H_2_O (0.0583 molL^− 1^), with pH set at 7.50 [55]. Roots and leaves were collected at 0 h, 3 h, 12 h and 48 h time points after salt-alkali stress treatment, as previously described by Magwanga et al. [56], in the determination of the role of proteins encoded by the *CYP450* genes in cotton under drought and salt stress conditions. Then, collected samples were used for transcriptome sequencing.

### Determination of malondialdehyde (MDA), proline (PRO) content and superoxide dismutase (SOD) activity

In order to detect the content of MDA and PRO and activity of SOD, leaves of MAR85, CRI12 and LAT40 were collected after 0 h, 3 h, 12 h and 48 h post to salt-alkali stress. The corresponding assay kits (Beijing Solarbio Science & Technology Co., Ltd.) were used for determining the content of MDA and PRO and activity of SOD.

### RNA extraction, cDNA library construction, and RNA-Seq

Total RNA was extracted from each cotton sample using TRlzol Reagent (Life technologies, California, USA) according to the instruction manual. RNA integrity and concentration were checked using an Agilent 2100 Bioanalyzer (Agilent Technologies, Inc., Santa Clara, CA, USA). mRNAs were isolated by NEBNext Poly (A) mRNA Magnetic Isolation Module (NEB, E7490). The cDNA libraries were constructed by following the manufacturer’s instructions of NEBNext Ultra RNA Library Prep Kit for Illumina (NEB, E7530) and NEBNext Multiplex Oligos for Illumina (NEB, E7500). Briefly, the enriched mRNA was fragmented into RNAs with approximately 200 nt, which were used to synthesize the first-strand cDNA and then the second cDNA. The double-stranded cDNAs were performed end-repair/dA-tail and adaptor ligation. The suitable fragments were isolated by Agencourt AMPure XP beads (Beckman Coulter, Inc.), and enriched by PCR amplification. Finally, the constructed cDNA libraries were sequenced on a flow cell using an Illumina HiSeq™ 2500 sequencing platform.

### Expression analysis

The row data of RNA-seq of M85, LAT40 and CRI12 were separately analyzed and the clean reads were obtained by removing reads containing adapter, reads containing ploy-N and lower quality reads from raw data. At the same time, Q_30_ [57], GC-content and sequence duplication level of the clean data were calculated. Raw sequences were transformed into clean reads after data processing. These clean reads were then mapped to the reference genome sequence. Only reads with a perfect match or one mismatch were further analyzed and annotated based on the reference genome. Tophat2 [58] tool soft was used to map with reference genome. Base on the reference genome, using Cufflinks software [59], mapped reads had been assembled. Quantification of gene expression levels was estimated by fragments per kilobase of transcript per million fragments mapped (FPKM). Base on mapped reads, using FPKM as the index, each gene was estimated by Cuffquant and Cufform. EBSeq software was used to identify the differential expression genes by Fold change (FC) *≥ 2* and *FDR ≤ 0.01* (False Discovery Rate). FDR was corrected using Benjamini-Hochberg method by *p*-value.

### Gene ontology (GO) and pathway enrichment analysis

Gene ontology enrichment analysis for differentially expressed gene sets was performed using agriGO v2.0 [60]. *P*-value for enrichment was calculated for each represented GO term. The GO terms exhibiting *P-value* of *≤0.001* and *FDR ≤ 0.05* were considered to be significantly enriched. Further, Kyoto Encyclopedia of Genes and Genomes (KEGG) pathway enrichment analysis of different sets of genes was performed using Cotton Functional Genomics Database (CottonFGD) (significance Q value ≤0.0001, Gene number for each analyzed term ≥3) [61]. The gene function annotation was performed by screening homolog genes of Arabidopsis (TAIR10) database (https://www.arabidopsis.org).

### Co-expression network analysis

The WGCNA [18, 30] package was used for gene co-expression network (GCN) analysis. Expression data profiles of different expressed genes were used for WGCNA. Based on log_2_ (FPKM) values, a matrix of Pearson’s correlation between all pair-genes were generated. Then, transformed into an adjacency matrix (a matrix of connection strengths) using the formula: connection strength (adjacency value) = Pearson’s correlation. Here, parameter represents soft threshold for the correlation matrix, which emphasize strong correlations between genes and penalize weak correlations [63]. A value of 12 was selected accurately selected by determination of soft-thresholding power and evaluation of scale free topology analysis. Next, the resulting adjacency matrix was transformed into a topological overlap matrix (TOM) via TOM similarity algorithm, and the genes were hierarchically clustered based on TOM similarity. The dynamic tree-cutting algorithm was used to cut the hierarchal clustering dendrogram and modules were defined after decomposing/combining branches to reach a stable number of clusters [64]. The module eigengene (ME) is defined as the first principal component of a given module. It can be considered a representative of the gene expression profiles in a module. Gene significance incorporates external information into the co-expression network. The higher the absolute value of GS, the more biologically significant is the gene. The value of ME and GS were obtained by WGCNA.

### Identification of tissue/stage-specific and tissue/sample-specific modules

We determined the correlation between each ME with the binary indicator (tissue/stage = 1 and all other samples = 0) as described in previous study [64]. Then, the association of module with tissue/stage-specific and sample were determined and the correlation matrix was draw by R package (ggplot). A positive correlation indicated that genes in a module were preferential expression in a particular tissue/stage or tissue/sample relative to all other samples.

### Candidate gene selection

*Hub* genes, highly interconnected with nodes in a module, have been considered functionally significant [63]. We used two different way for screening *hub* genes: (1) According to “network Screening” function of WGCNA package, possibility weight (p. weight) value of each gene was obtained based on gene significance (GS) and modular membership (MM). The lower p. weight value means that the gene is higher correlation with traits (treatment stages). Top 30 hub genes were identified as hub genes based on p. weight; (2) Base one TOM of all different expression genes, top 150 highly connected genes and the top 300 connections of highly connected genes for higher correlation modules of different stages were selected for analysis. Then, top 30 genes that were central and highly connected were identified by visualized using the Cytoscape 3.3.0 software.

### RT-qPCR analysis

Results of RNA-seq were validated via RT-qPCR experiments. Real-time PCR analyses were performed as the user manual of the TransScript II All-in-One First-Strand cDNA Synthesis SuperMix for PCR (TransGen Biotech) and the SYBR Premix Ex Taq II kit (Roche) described. The housekeeping gene was *GhActin*. The gene-specific primers designed using Primer-BLAST (http://www.ncbi.nlm.nih.gov/tools/primer-blast/) tool and primers are listed in Additional file 5: Table S1, Additional file 6: Table S2, Additional file 7: Table S3, Additional file 8: Table S4, Additional file 9: Table S5, Additional file 10: Table S6, Additional file 11: Table S7, Additional file 12: Table S8. The experiments of real-time PCR were performed using three biological replicates for each tissue sample and at least three technical replicates of each biological replicate. The value of genes folds change was calculated using the 2^-ΔΔCT^ method [65].

### Virus induced gene silencing (VIGS) of candidate genes, *GhSOS3 (Gh_A01G1607)* and *GhCBL10 (Gh_D05G0440)*, in *G. hirsutum* race marie-galante 85

The TRV2 (tobacco rattle virus) vector constructs pYL156 and pYL192 (pYL156-Ctrl, pYL156-PDS, pYL156-*GhCBL10*, pYL156-*GhSOS3*, pYL156-GhCBL10) were prepared and introduced into Agrobacterium tumefaciens strain GV4104. In order to monitor the silencing efficiency, the pYL156-PDS vector was constructed as a visual marker. Primers used to generate TRV vector were listed in Additional file 5: Table S1, Additional file 6: Table S2, Additional file 7: Table S3, Additional file 8: Table S4, Additional file 9: Table S5, Additional file 10: Table S6, Additional file 11: Table S7, Additional file 12: Table S8. The agrobacterium culture was agroinfiltrated into two expanded cotyledons of 10-day-old soil-grown seedling of *G. hirsutum* race marie-galante 85 (MAR85). The cotton seedlings were planted in a 26 °C and 16 h light/8 h dark cycle. At least 24 seedlings were inoculated for each construct. At 14 days after agrobacterium inoculation when VIGS was established, the silenced seedlings were posted to salt-alkali treatment. The phenotype was shown in Fig. 7. At 20 days of salt-alkali stress treatment, the leaf samples were collected for expressed level, MDA, PRO and SOD assay.

### Statistical analysis and graphic presentation

Statistical analysis of the experimental data was statistically analyzed using the R package. Prcomp package was used for Principal components analysis (PCA) analysis and Graphic presentation was performed using scatterplot3d package. Graphic presentation of expression level distribution was performed using boxplot package. The hierarchical Clustering package was used for cluster analysis. Graphic presentation of cluster tree and heatmap were performed using gplots package. Graphic presentations of network/module construction were performed using WGCNA package.

## Supplementary information

**Additional file 1: Figure S1.** Different expression genes (DEGs) annotation via the GO database. Agri GO 2.0 analysis of DEGs in leaf and root. Each box shows the GO term number, the *p*-value in parenthesis, and GO term. The pair of numerals on the left represents the number of genes in input list associated with that GO term and number of genes in the input list. The pair of numerals in the right represents the number of genes associated with a particular GO term in the Gossypium database and the total number of Gossypium genes with GO annotations in the Gossypium database. Box colors indicate levels of statistical significance: yellow = 0.05; orange = e− 5; and red = e− 9. The plates A and B depict the significant enrichment GO terms of leaf and root sample DEGs, respectively.

**Additional file 2: Figure S2.** Different expression genes (DEGs) annotation via Kyoto Encyclopedia of Genes and Genomes (KEGG) database. Plots A and B depict, respectively, the significant enrichment KEGG terms of L3 h, L12 h, L48 h, R3h, R12h and R48h DEGs, respectively.

**Additional file 3: Figure S3.** Determination of soft-thresholding power in the weighted gene co-expression network analysis (WGCNA) and evaluation of scale free topology. (A) Analysis of the scale-free fit index for various soft-thresholding powers (β). (B) Analysis of the mean connectivity for various soft-thresholding power. (C) A histogram of network connectivity when β = 12; (D) A log-log plot of the same histogram when β = 12. The approximate straight-line relationship (high R2 value) shows the approximate scale free topology.

**Additional file 4: Figure S4.** The heatmap plot of the topological overlap matrix. In the heatmap, rows and columns correspond to single genes, lighter colors represent low topological overlap, and progressively darker orange and red colors represent higher topological overlap. The corresponding gene dendrograms and module assignment are shown on the left and top.

**Additional file 5: Table S1**. Gene ontology (GO) enrichment analysis of DEGs in leaf.

**Additional file 6: Table S2.** Gene ontology (GO) enrichment analysis of DEGs in root.

**Additional file 7: Table S3.** Kyoto Encyclopedia of Genes and Genomes (KEGG) enrichment of DEGs in leaf.

**Additional file 8: Table S4.** Kyoto Encyclopedia of Genes and Genomes (KEGG) enrichment of DEGs in root.

**Additional file 9: Table S5.** Number of members of different TF families showing in the upland cotton at different stages after alkali-salt stress treatment.

**Additional file 10: Table S6.** The hub genes of each tissue/stage-specific module.

**Additional file 11: Table S7.** GO enrichment of each tissue/stage-specific modules.

**Additional file 12: Table S8.** List of primers used for RT-qPCR and VIGS.

## Data Availability

All the relevant data and supplementary data are all availed. All supplementary data supporting this research work are all made available in a public data repository and can be accessed through the link https://www.ncbi.nlm.nih.gov/sra/PRJNA531727.

## Abbreviations

AMPK: AMP-activated protein kinase
BLAST: Basic Local Alignment Search Tool
BP: Biological process
CBL: Calcineurin B-like
CC: Cellular component
DEG: Differentially expressed genes
FAO: Food and Agriculture Organization
FC: Fold change
FDR: False discovery rate
FPKM: Fragments per kilobase of transcript per million fragments mapped
GC: Guanine Cytosine
GCN: Gene co-expression network
GO: Gene ontology
GS: Gene significance
KEGG: Kyoto Encyclopedia of Genes and Genomes
LEA: Late embryogenesis abundant
MATE: Multidrug and toxic compound extrusion
MDA: Malondialdehyde
ME: Module eigengene
MF: Molecular functions
MM: Modular membership
NGS: Next generation sequencing
PCA: Principal components analysis
PCR: Polymerase chain reaction
ROS: Reactive oxygen species
SOD: Superoxide dismutase
SOS: Salt overly sensitive
TOM: Topological overlap matrix
UNESCO: United Nations Educational, Scientific and Cultural Organization
VIGS: Virus induced gene silencing
WGCNA: Weighted correlation network analysis
