# Supplementary figures and images for: Genetic regulatory networks for salt-alkali stress in Gossypium hirsutum with differing morphological characteristics
Source: BMC Genomics. 2020 Jan 6;21:15. doi: 10.1186/s12864-019-6375-9 (PMC6945603; doi:10.1186/s12864-019-6375-9)

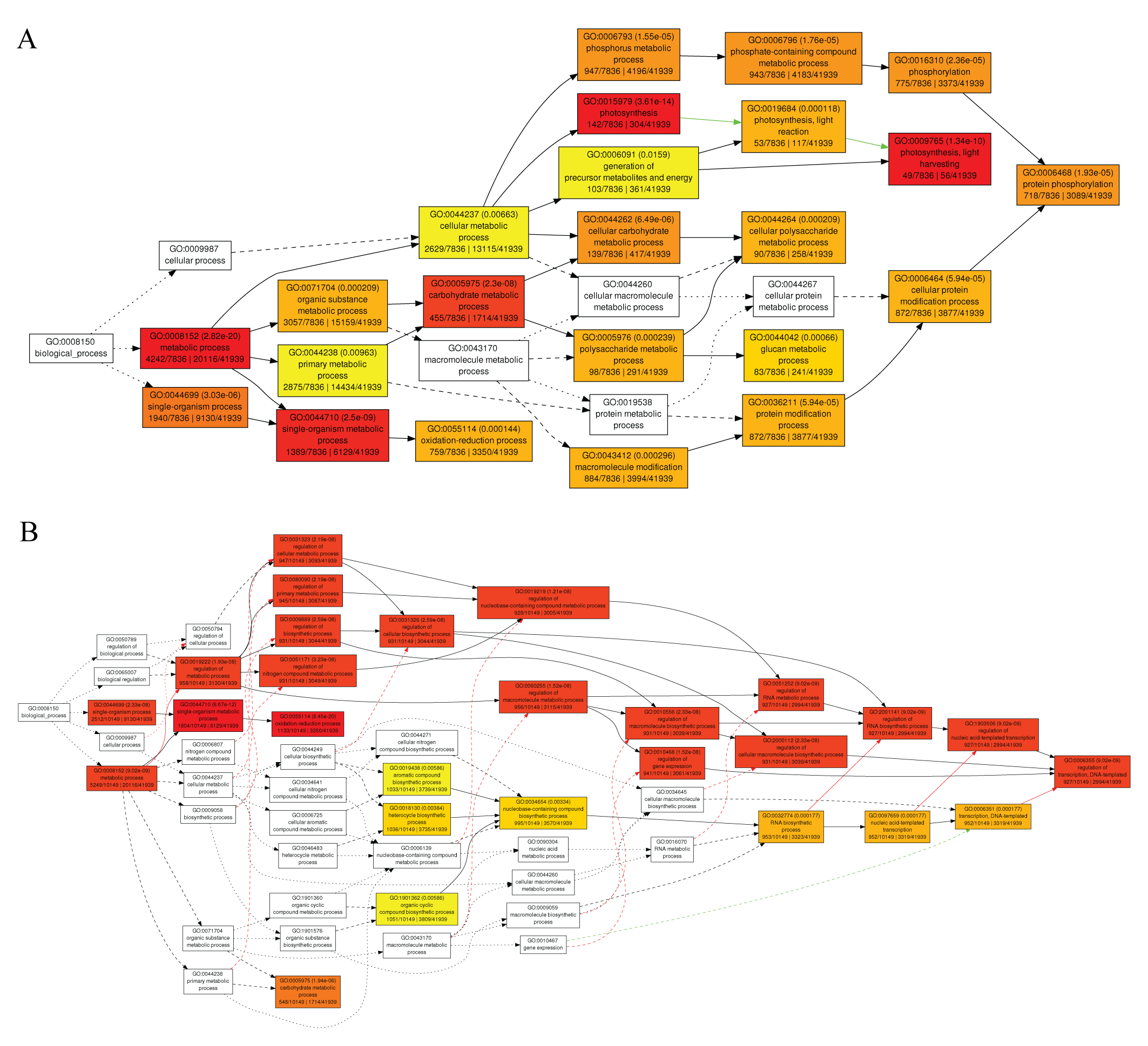

Supplement: Supplementary file 1 — Additional file 1: Figure S1. Different expression genes (DEGs) annotation via the GO database. Agri GO 2.0 analysis of DEGs in leaf and root. Each box shows the GO term number, the p-value in parenthesis, and GO term. The pair of numerals on the left represents the number of genes in input list associated with that GO term and number of genes in the input list. The pair of numerals in the right represents the number of genes associated with a particular GO term in the Gossypium database and the total number of Gossypium genes with GO annotations in the Gossypium database. Box colors indicate levels of statistical significance: yellow = 0.05; orange = e− 5; and red = e− 9. The plates A and B depict the significant enrichment GO terms of leaf and root sample DEGs, respectively. [file 12864_2019_6375_MOESM1_ESM.tif]

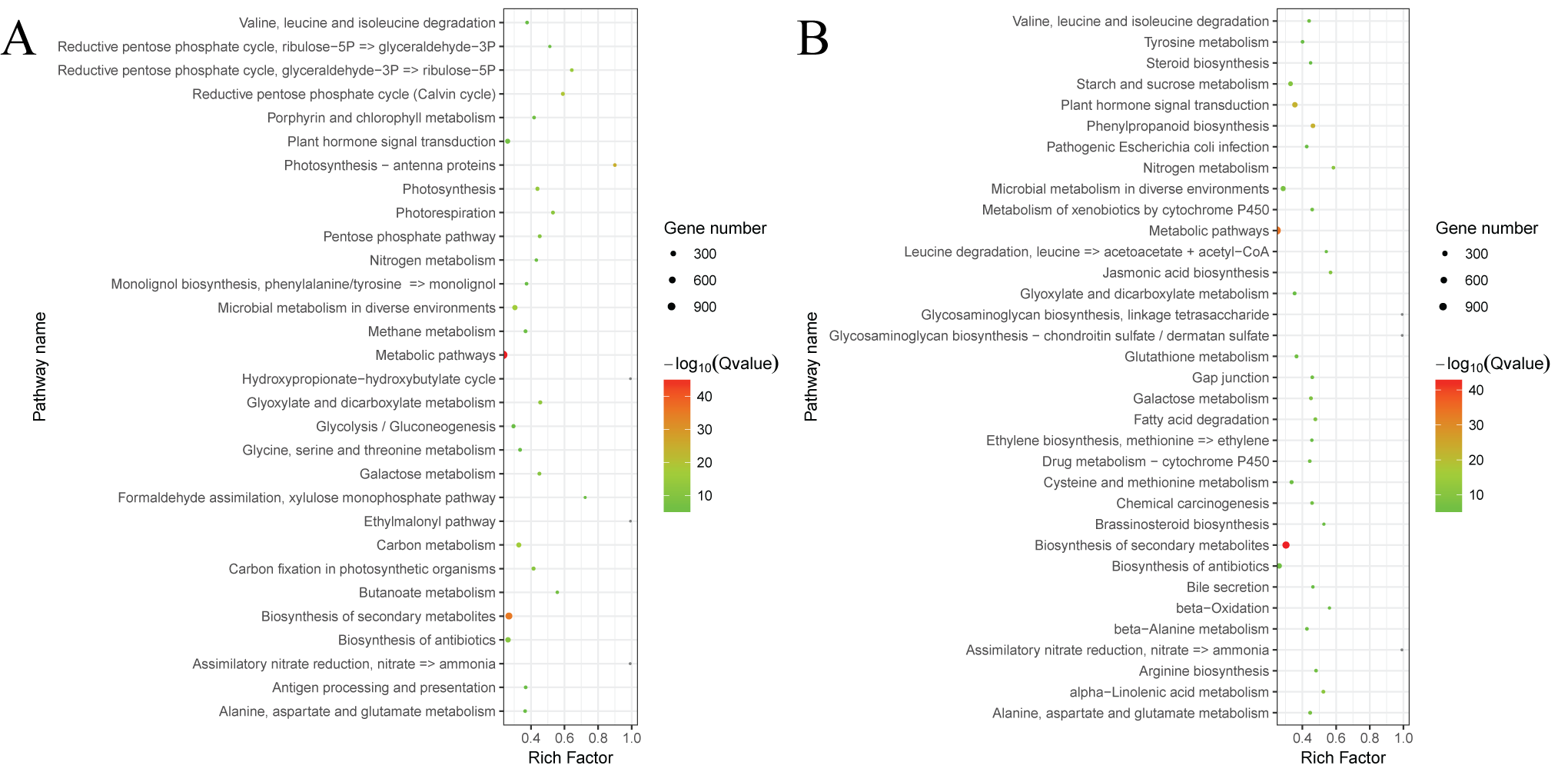

Supplement: Supplementary file 2 — Additional file 2: Figure S2. Different expression genes (DEGs) annotation via Kyoto Encyclopedia of Genes and Genomes (KEGG) database. Plots A and B depict, respectively, the significant enrichment KEGG terms of L3 h, L12 h, L48 h, R3h, R12h and R48h DEGs, respectively. [file 12864_2019_6375_MOESM2_ESM.tif]

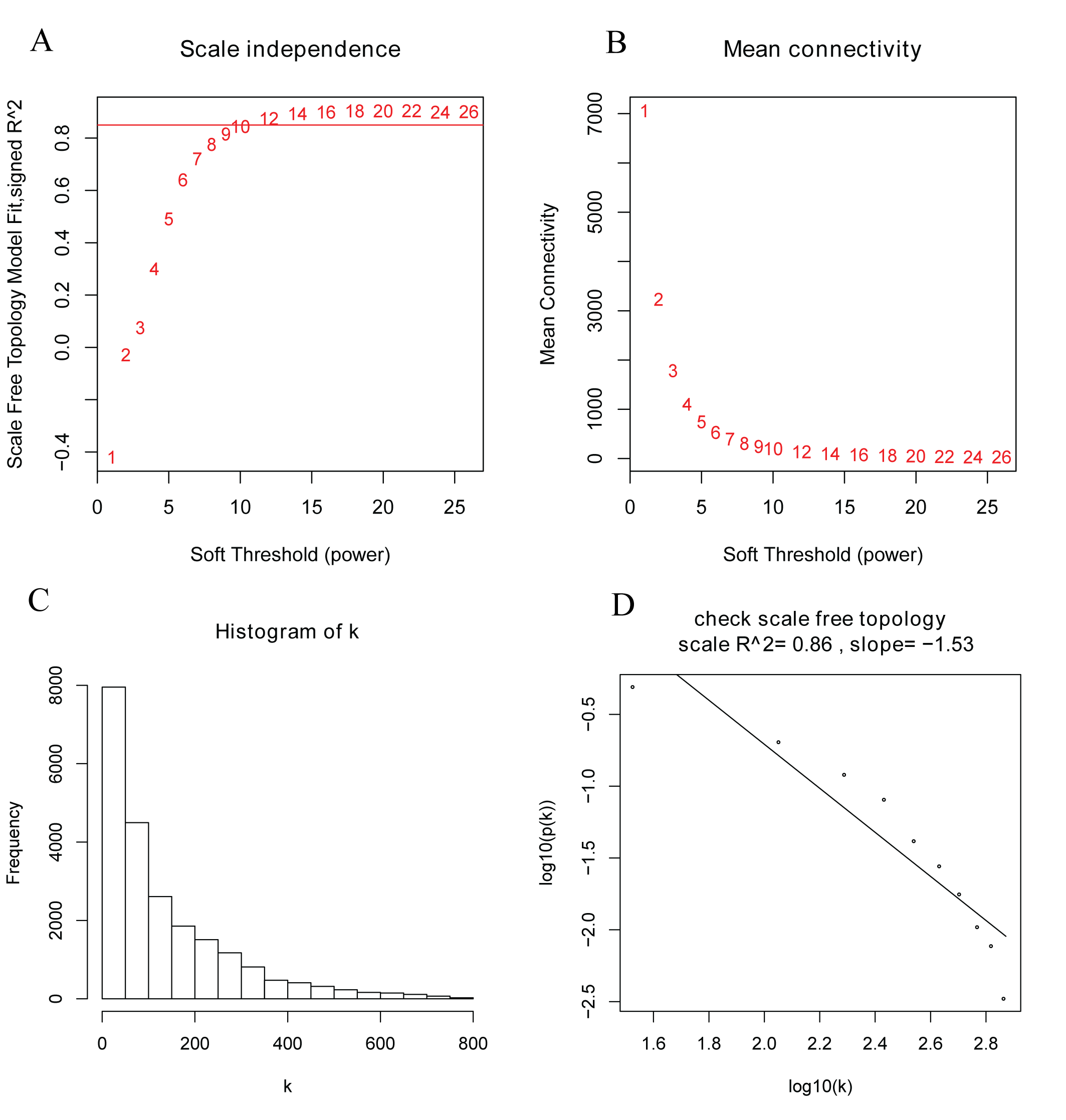

Supplement: Supplementary file 3 — Additional file 3: Figure S3. Determination of soft-thresholding power in the weighted gene co-expression network analysis (WGCNA) and evaluation of scale free topology. (A) Analysis of the scale-free fit index for various soft-thresholding powers (β). (B) Analysis of the mean connectivity for various soft-thresholding power. (C) A histogram of network connectivity when β = 12; (D) A log-log plot of the same histogram when β = 12. The approximate straight-line relationship (high R2 value) shows the approximate scale free topology. [file 12864_2019_6375_MOESM3_ESM.tif]

Network heatmap plot, selected genes

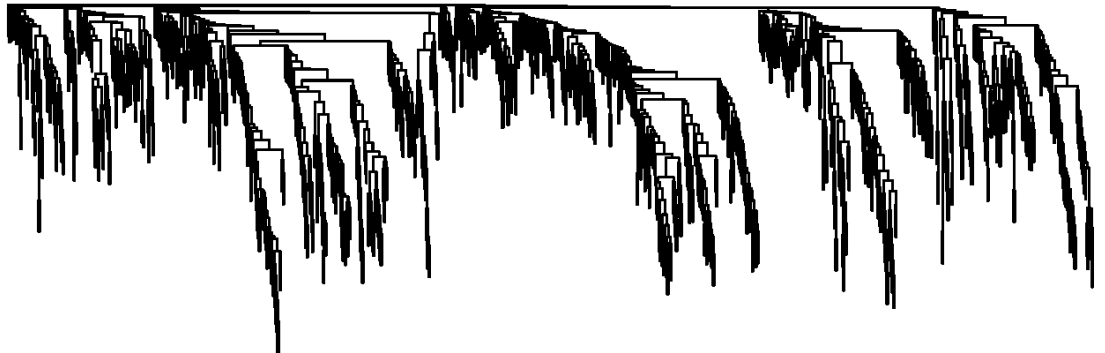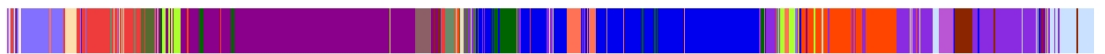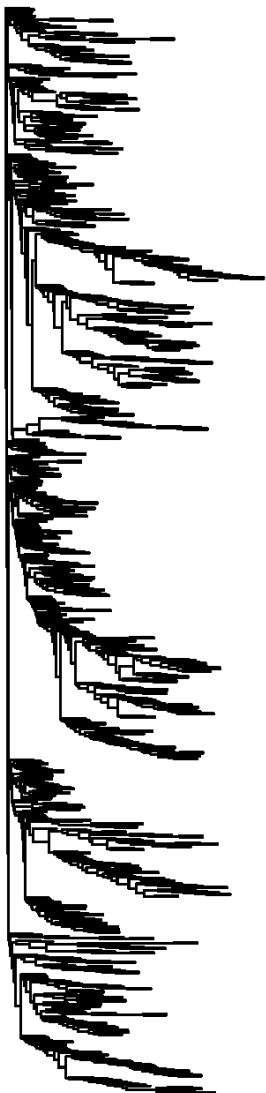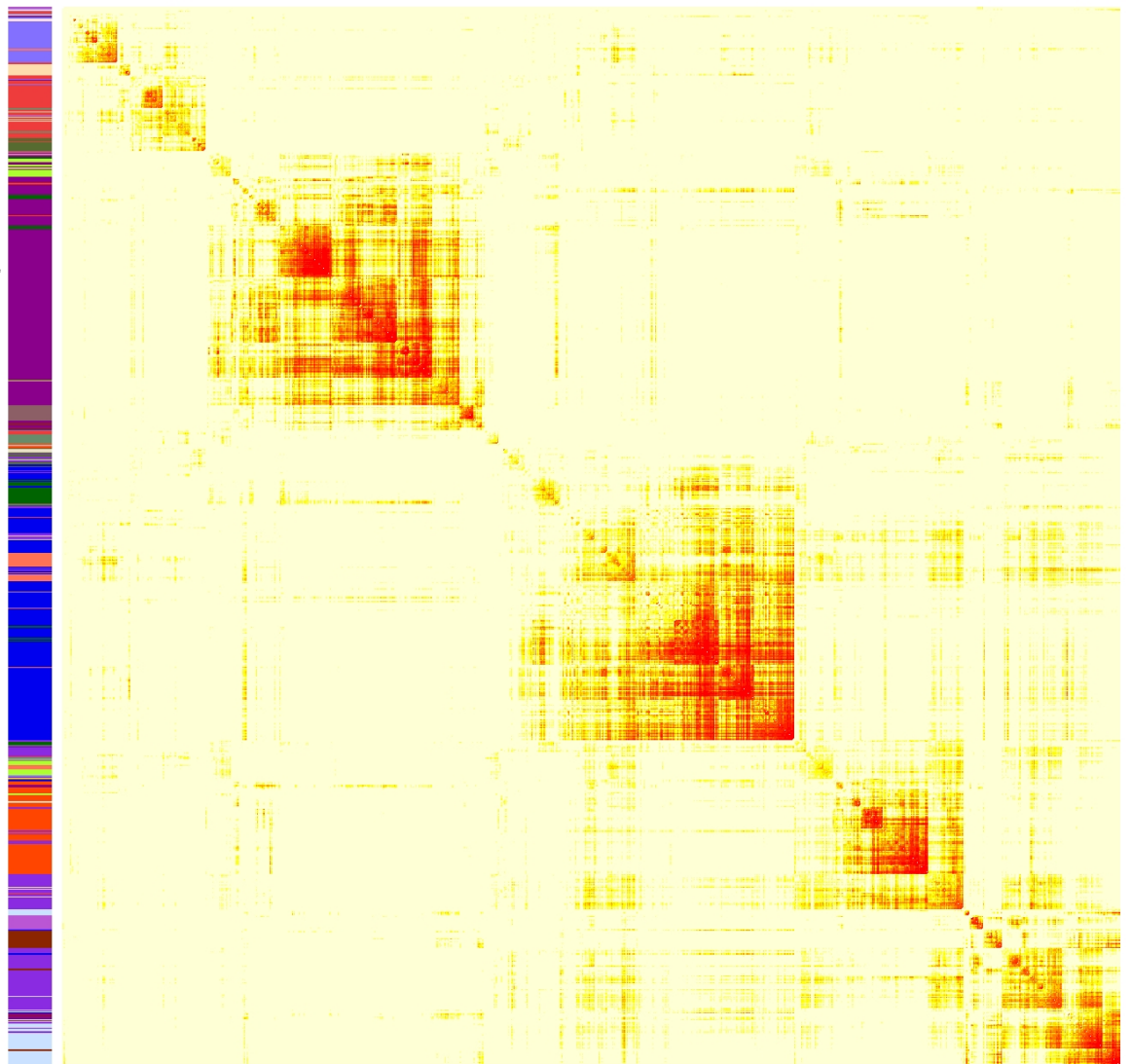

Supplement: Supplementary file 4 — Additional file 4: Figure S4. The heatmap plot of the topological overlap matrix. In the heatmap, rows and columns correspond to single genes, lighter colors represent low topological overlap, and progressively darker orange and red colors represent higher topological overlap. The corresponding gene dendrograms and module assignment are shown on the left and top. [file 12864_2019_6375_MOESM4_ESM.pdf]
